# Analysis of gene network robustness based on saturated fixed point attractors

**DOI:** 10.1186/1687-4153-2014-4

**Published:** 2014-03-20

**Authors:** Genyuan Li, Herschel Rabitz

**Affiliations:** 1 Department of Chemistry, Princeton University, Princeton, NJ 08544, USA

**Keywords:** Robustness of gene networks, Fixed point attractor, Sigmoidal function, Yeast cell-cycle network

## Abstract

The analysis of gene network robustness to noise and mutation is important for fundamental and practical reasons. Robustness refers to the stability of the equilibrium expression state of a gene network to variations of the initial expression state and network topology. Numerical simulation of these variations is commonly used for the assessment of robustness. Since there exists a great number of possible gene network topologies and initial states, even millions of simulations may be still too small to give reliable results. When the initial and equilibrium expression states are restricted to being saturated (i.e., their elements can only take values 1 or −1 corresponding to maximum activation and maximum repression of genes), an analytical gene network robustness assessment is possible. We present this analytical treatment based on determination of the saturated fixed point attractors for sigmoidal function models. The analysis can determine (a) for a given network, which and how many saturated equilibrium states exist and which and how many saturated initial states converge to each of these saturated equilibrium states and (b) for a given saturated equilibrium state or a given pair of saturated equilibrium and initial states, which and how many gene networks, referred to as viable, share this saturated equilibrium state or the pair of saturated equilibrium and initial states. We also show that the viable networks sharing a given saturated equilibrium state must follow certain patterns. These capabilities of the analytical treatment make it possible to properly define and accurately determine robustness to noise and mutation for gene networks. Previous network research conclusions drawn from performing millions of simulations follow directly from the results of our analytical treatment. Furthermore, the analytical results provide criteria for the identification of model validity and suggest modified models of gene network dynamics. The yeast cell-cycle network is used as an illustration of the practical application of this analytical treatment.

## 1 Introduction

A subset of genes in a cell whose protein products mutually regulate one another’s expression at the transcriptional level will be referred to as a ‘gene network’ in this paper. The concentration of proteins encoded by the genes changes in time due to auto- and cross-regulation by the gene products. Each of such network is considered as a dynamical system. Gene networks must be robust with respect to ever-changing environments. Robustness here refers to the ability of a gene network to respond to short-term changes in the environment and quickly return to its functional steady state. Moreover, a gene network itself may endure small structural changes and mutations, while still retaining its desired steady state. The robustness of gene networks depends on their topology with some networks being more stable than others. The analysis of the relationship between the topology of a gene network and its robustness to environmental and structural perturbations is important both theoretically and practically [1-10].

Recently, Wagner and coworkers considered the robustness of gene networks [11-14], and a similar assessment was given by Cho et al. [15] In these works, a simplified model proposed by Wagner was used to describe the dynamics of the gene expression states [11,12]. Let **G**=(*G*_1_,…,*G*_*n*_) represent the *n* genes in a network. The concentration of proteins encoded by the genes (*G*_1_,…,*G*_*n*_) is denoted by **P**=(*P*_1_,…,*P*_*n*_). For computational convenience, the admissible concentration range for each *P*_*i*_ is normalized and restricted to the interval [0,1], where *P*_*i*_=1 corresponds to the maximum possible concentration, i.e., the corresponding gene *G*_*i*_ is in a state of maximum transcriptional activation. It is also assumed that *P*_*i*_=0.5 means that gene *i* is 50% ‘on’. The dynamics of the expression states of the genes in a network is often described by some sigmoidal function *g*_*c*_(*x*)

(1) $$P_{i}(t+\tau )=g_{c}\left(\overset{n}{\underset{j=1}{\sum }}w_{\text{ij}}P_{j}(t)\right),\;(i=1,2,\ldots ,n)$$

where *τ* is a time constant characteristic of the process under consideration. In some work, *τ* was set to be 1. The constant *w*_*ij*_∈ℜ describes the strength of interaction (i.e., transcriptional regulation) of the product of gene *j* with gene *i*, i.e., the degree of transcriptional activation (*w*_*ij*_>0), repression (*w*_*ij*_<0), or absence (*w*_*ij*_=0). These constants define a matrix of connectivities *W*=(*w*_*ij*_) within the network. To facilitate the analytical treatment, the variable transformation

(2) $$\text{S}=2\text{P}-{\left(1\ldots 1\right)}^{T}$$

is employed. Using the sigmoidal function *σ* proposed by Siegal [16] and Cho [15] for *g*_*c*_, (1) becomes

(3) $$\begin{array}{ll} \;S_{i}(t+\tau ) & =\sigma \left(\overset{n}{\underset{j=1}{\sum }}w_{\text{ij}}S_{j}(t)\right)\\ & =\frac{2}{1+\exp\left[-\overset{n}{\underset{j=1}{\sum }}w_{\text{ij}}S_{j}(t)\right]}-1,\;(i=1,2,\ldots ,n) \end{array}$$

with *S*_*i*_∈[−1,1], and *S*_*i*_=0 corresponding to 50% of gene *i* being ‘on’. Notwithstanding the simplicity of (3), variants of this model have been successfully used to study (a) the robustness of gene regulatory networks [12,16,17], (b) the role of robustness in evolutionary innovation [18,19], and (c) how recombination can produce negative epistasis [20].

Mjolsness at al. [21] proposed a model

(4) $$\begin{array}{c} \;\tau_{a}\frac{{\text{dv}}_{i}^{a}}{\text{dt}}=g_{a}\left(\overset{n}{\underset{b=1}{\sum }}T^{\text{ab}}v_{i}^{b}+h^{a}\right)-\lambda_{a}v_{i}^{a},\;(a=1,2,\ldots ,n) \end{array}$$

to describe the dynamics for each element of primitive objects **v**_*i*_ (cells, nuclei, fibers, and synapses), where *g*_*a*_ is a sigmoidal threshold function, *T*^*ab*^ is similar to *w*_*ij*_ in (3); $v_{i}^{a},v_{i}^{b}$ denote the elements of vector **v**_*i*_; *h*^*a*^ determines the threshold of *g*_*a*_. The long-time behavior of this system has been studied, and, in some cases, is controlled by a simple limit set

(5) $$v_{i}^{a}=\frac{1}{\lambda_{a}}g_{a}\left(\overset{n}{\underset{b=1}{\sum }}T^{\text{ab}}v_{i}^{b}+h^{a}\right),$$

which is similar to (3) with the additional parameters *λ*_*a*_ and *h*^*a*^. This model has been successfully applied to treat the blastoderm of *Drosophila melanogaster*[21].

Similarly, Mendoza and Alvarez-Buylla [22] used a model

(6) $$x_{i}(t+1)=H\left(\overset{n}{\underset{j=1}{\sum }}w_{\text{ij}}x_{j}(t)-\theta_{i}\right),$$

where *H* is the Heaviside step function

(7) $$H(x)=\left\{\begin{array}{cc} 1, & \text{if}\;x>0,\\ 0, & \text{if}\;x\leq 0 \end{array}\right)$$

to describe the dynamics of a genetic regulatory network for *Arabidopsis thaliana* flower morphogenesis. This model is also similar to (3) except that the sigmoidal function *σ* is replaced by the Heaviside step function and a threshold parameter *θ*_*i*_ is included.

All these models present simplified descriptions of gene network dynamics. Nevertheless, the models are still useful for obtaining insights into the dynamics of gene networks. In the following analysis for gene network robustness, we will employ the sigmoidal function model in (3), and its modification with threshold parameters.

The robustness of a gene network specified by *W* to noise (environmental) and mutation (structural) perturbations may be expressed as the stability of the final equilibrium (or steady) expression state **S**(*∞*) obtained from the solution of (3), respectively, to changes of initial expression state **S**(0) and to changes of *W*. A complete and reliable robustness analysis would seem to call for an exhaustive sampling over the space of possible initial states for a given network and then repeating the same simulations for all possible networks. This task is infeasible as there are many possible networks *W*, and each *W* may have many initial/final equilibrium expression states. Consider a simple case where *S*_*i*_(0) and *S*_*i*_(*∞*)(*i*=1,2,…,*n*) can only take values −1,1 and *w*_*ij*_ can only take values −1, 0, 1. In this case, there are 2^*n*^ possible initial/final states and $3^{n^{2}}$ possible gene networks. For a modest network of size *n*=20, there are 2^20^=1,048,576 initial/final expression states and 3^400^≈7×10^190^ gene networks. Even if one arbitrarily makes the restriction that 75% of the *w*_*ij*_ interactions are zero, and that the remaining 25% of the *w*_*ij*_ interactions can only take nonzero values −1,1 to reduce the possible number of networks [13], for *n*=20 there are still $C_{400}^{100}\times 2^{100}$ possible gene networks. Further restriction may also be applied to reduce the possible number of initial expression states [13]. Even under these restrictions, solving (3) for all possible initial expression states and gene networks is still infeasible, and only a small fraction of them can be randomly sampled and simulated. Such limitations leave open the reliability of the conclusions obtained from the simulations.

Previous work [13] concerned networks with connectivity *W* whose expression dynamics start from a prespecified initial state **S**(0) at some time *t*=0 and arrive at a prespecified stable equilibrium or ‘target’ expression state **S**(*∞*); these networks are referred to as ‘viable’. Then, the values of some elements of **S**(0) or *W* were changed for each viable network to check whether **S**(*∞*) is reached. These studies entailed performing millions of simulations with different network topologies and initial expression states. Although the number of simulations is much smaller than 2^*n*^ and $3^{n^{2}}$ for *n*=20, some specific conclusions were obtained. First, the fraction of viable networks, that is, networks that arrive at a prespecified target expression state **S**(*∞*) given an initial gene expression state **S**(0) to the total number of possible networks, is generally very small. For moderately sized networks of *n*=20 genes (with the number *M* of nonzero *w*_*ij*_ set to be 200, and the fraction *d* of elements different between **S**(0) and **S**(*∞*) set to 0.5), the fraction of viable networks was found to be *v*_*f*_=5.1×10^−9^±1.7×10^−10^. Due to the large numbers 2^*n*^ and $3^{n^{2}}$, the qualitative correctness of this conclusion is clear. Since there are 2^*n*^ possible equilibrium states, even if we only consider the factor of expression states, the probability that a network *W* arrives at a prespecified **S**(*∞*) is expected on the order of 1/2^*n*^. The viable networks in this prior work could be organized as a graph with each node corresponding to a network of a given topology, and two nodes are connected by an edge if they differ by a single regulatory interaction (i.e., they differ in one element *w*_*ij*_). Remarkably, this graph is connected and can be easily traversed by gradual changes of the network topology. Thus, highly robust topologies can evolve from topologies with low robustness through gradual Darwinian topological changes. These results are claimed to be valid for discrete and continuous *w*_*ij*_ taking values [−1,0,1] and over the interval [−*a*,*a*], respectively. While simulations are valuable, they do not provide a complete picture.

Ciliberti et al. [13] considered the case where each element of the initial and equilibrium expression states, **S**(0) and **S**(*∞*), can only take the values 1 or −1 corresponding to maximum and minimum possible protein concentrations. We call them *saturated* expression states. Under this condition, the present paper provides an analytical robustness assessment of gene networks whose dynamics can be described by (3) and its modification with threshold parameters. This analysis can determine (a) for a given network, which and how many saturated equilibrium states exist, and which and how many saturated initial states converge to each of these saturated equilibrium states; (b) for a given saturated equilibrium state, or a given pair of saturated equilibrium and initial states, which and how many gene networks, referred to as viable, share this saturated equilibrium state or the pair of saturated equilibrium and initial states. We also show that the viable networks sharing a given saturated equilibrium state must follow certain patterns. These capabilities of the analytical treatment make it possible to properly define and accurately determine robustness to noise and mutation for gene networks. Previous network research conclusions drawn from performing millions of simulations follow directly from the results of our analytical treatment. Furthermore, the analytical results provide criteria for identification of model validity and suggest modified models of gene network dynamics.

The paper is organized as follows: Section 2 first defines the saturated state and saturated fixed point attractor for dynamics (3), and then gives the necessary and sufficient condition for a gene network to have a given saturated equilibrium state. Sections 3 and 4 analyze the robustness to noise and mutation, respectively. Section 5 proposes a modification of dynamics (3) with threshold parameters. Section 6 gives an illustration of the practical application of this analytical treatment: the model construction of the yeast cell-cycle network and its robustness assessment. The details of the treatment of the yeast cell-cycle network are given in an Additional file 1: Supplementary information. Finally, Section 7 presents conclusions. Mathematical proofs of the theorems in the main text are given in the Appendix.

## 2 Saturated states and fixed point attractors

In this work, the initial and equilibrium expression states *S*_*i*_(0) and *S*_*i*_(*∞*) for gene *i* can only be either active (*S*_*i*_(0) and *S*_*i*_(*∞*)=1) or inactive (*S*_*i*_(0) and *S*_*i*_(*∞*)=−1) [11,12,14]. The initial and equilibrium expression states with *S*_*i*_(0) and *S*_*i*_(*∞*)=±1 are referred to as *saturated* initial and equilibrium expression states. *Under the condition that the initial and equilibrium expression states****S***(0)*and****S***(*∞*)*are saturated, we may analyze the robustness and evolvability of gene networks analytically*, as explained below.

### 2.1 Saturated sigmoidal function

A continuous function *f*(*x*) defined on ℜ satisfying **(f1)***f*(*x*)=1 if *x*≥1, *f*(*x*)=−1 if *x*≤−1, **(f2)***f*(*x*) is a strictly increasing and continuous function for *x*∈[−1,1] and *f*(0)=0,

is called a saturated sigmoidal function. Furthermore, if **(f3)***f*(*x*)≥*x* for *x*∈(0,1] and *f*(*x*)≤*x* for *x*∈[−1,0),

we call *f*(*x*)] a dissipative saturated sigmoidal function.

**Figure 1:**
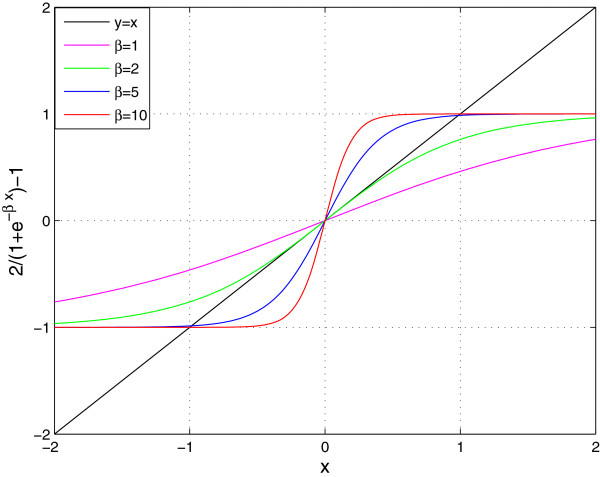
**The function** ***σ***_***β***_**(*x*).**

Note that for the particular sigmoidal function *σ*_*β*_(*x*) in domain [-1, 1]

(8) $$x(t+\tau )=\sigma_{\beta }(x(t))=\frac{2}{1+e^{-\mathrm{\beta x}(t)}}-1,$$

the conditions (**f1**) to (**f3**) are approximately satisfied when *β* is sufficiently large. For example, when *β*=5 and 10, we have *σ*_*β*_(1)=0.9866 and 0.9999 (approximately 1); and *σ*_*β*_(−1)=−0.9866 and −0.9999 (approximately −1), respectively. Therefore, in numerical simulation, *σ*_*β*_(*x*) can be considered as a dissipative saturated sigmoidal function for a sufficiently large *β*. We refer to *β*=5 and 10 as having 0.99 and 0.9999 confidence levels for the dissipative saturated sigmoidal function (8), because |*σ*_*β*_(±1)| is equal to 0.99 and 0.9999, respectively (see Figure 1). In the sequel, we set *β*≥5.

### 2.2 Necessary and sufficient condition for a saturated state **S** to be an equilibrium state or a fixed point attractor of dynamics (3) with a given *W*

When the equilibrium states **S**(*∞*) are saturated, the analysis of robustness and evolvability for gene networks can be readily performed by utilizing Feng and Tirozzi’s treatment for neural networks [23].

#### 

**Definition1**. A saturated state **S** in [−1,1]^*n*^ is called a saturated fixed point attractor (or saturated equilibrium state) of dynamics (3), if there exists a nonempty neighborhood *B*(**S**) of **S** such that

$$\lim_{t\to \infty }\text{S}(t)=\text{S}$$

for **S**(0)∈*B*(**S**) and $\overset{n}{\underset{j=1}{\sum }}w_{\text{ij}}S_{j}\neq 0$, for all *i*.

It is easy to see that a saturated state **S** is a saturated fixed point of dynamics (3) with 0.99 (*β*=5) or 0.9999 (*β*=10) confidence level when

(9) $$\overset{n}{\underset{j=1}{\sum }}w_{\text{ij}}\;\text{sign}(S_{j})\geq \beta ,\;\text{if}\;\;S_{i}=1,$$

(10) $$\overset{n}{\underset{j=1}{\sum }}w_{\text{ij}}\;\text{sign}(S_{j})\leq -\beta ,\;\;\;\;\;\text{if}\;\;S_{i}=-1,$$

and

(11) $$\lim_{t\to \infty }S_{i}(t)\approx \left\{\begin{array}{cc} 1, & \text{if}\;\;S_{i}=1\\ -1, & \text{if}\;\;S_{i}=-1 \end{array}\right)\;(i=1,2,\ldots ,n),$$

i.e., **S** is a saturated fixed point with 0.99 (*β*=5) or 0.9999 (*β*=10) confidence level. When

$$\left|\overset{n}{\underset{j=1}{\sum }}w_{\text{ij}}\;\mathrm{sign}(S_{j})\right|<5,$$

for any *i*, then −0.99<*S*_*i*_(*t*+*τ*)<0.99 for any *t*, and the gene network cannot have a saturated fixed point.

Let **S** be a saturated state. We define

(12) $$J^{+}(\text{S})=\{i,S_{i}=1\},\;J^{-}(\text{S})=\{i,S_{i}=-1\}$$

which denote, respectively, the two sets of all integers in {1,2,…,*n*} with *S*_*i*_=1 and *S*_*i*_=−1.

#### 

**Theorem1**. The necessary and sufficient condition for a saturated state **S** to be an equilibrium expression state or a fixed point attractor of dynamics (3) with a given matrix *W* is

(13) $$\underset{j\in J^{+}(\text{S})}{\sum }w_{\text{ij}}-\underset{j\in J^{-}(\text{S})}{\sum }w_{\text{ij}}\geq \beta ,\;\text{if}\;\;i\in J^{+}(\text{S}),$$

(14) $$\underset{j\in J^{+}(\text{S})}{\sum }w_{\text{ij}}-\underset{j\in J^{-}(\text{S})}{\sum }w_{\text{ij}}\leq -\beta ,\;\text{if}\;\;i\in J^{-}(\text{S}),$$

or equivalently

(15) $$S_{i}\left(\overset{n}{\underset{j=1}{\sum }}w_{\text{ij}}S_{j}\right)\geq \beta ,\;(i=1,2,\ldots ,n).$$

#### 

*Proof*. If a saturated state **S** is a fixed point of (3), it must satisfy (9,10), i.e., (13,14). If (13,14) are satisfied, so are (9,10), then (11) is satisfied and **S** is a fixed point. A saturated state **S** satisfying (13,14) (or equivalently (15)) is a fixed point attractor. The proof is given in Theorem A2 in the Appendix. □

There are a total 2^*n*^ saturated states **S**. Since (15) only involves simple multiplication and summation, for a modest *n* one can test all 2^*n*^ saturated vectors **S** for a given *W* and find all of its saturated fixed point attractors without iteratively solving the sigmoidal function (3). For *n*=11 and 2^11^=2,048, the test takes 0.01 s by Matlab on a Dell Precision Workstation T3400.

#### 

**Figure 2:**
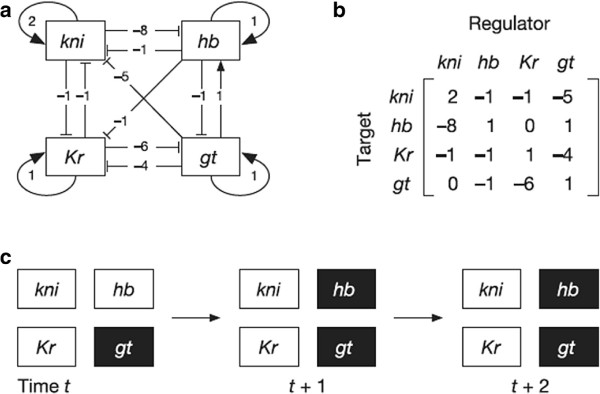
**The network model for the gap gene system of*Drosophila melanogaster* (reproduced from **[20]**).(a)** Network representation of the regulatory interactions between four gap genes (*gt*, giant; *hb*, hunchback; *kni*, knirps; *Kr*, Krüppel). **(b)** Corresponding matrix *W*. **(c)** graphic representation of the gene expression states of each gap gene over three successive time steps of a sigmoidal function similar to (3) where a filled box denotes ‘on’ (1), and an open box denotes ‘off’ (−1).

**Example1**. Consider the network model proposed by Azevedo et al. [20] for the gap gene system of *Drosophila melanogaster* shown in Figure 2.

The authors obtained the equilibrium state **S**_1_=(−11−11) by iteratively solving a sigmoidal function similar to (3). Using (9,10), we can determine this solution by noting

$$W\cdot {\text{S}}_{1}=\left[\begin{array}{cccc} 2 & -1 & -1 & -5\\ -8 & 1 & 0 & 1\\ -1 & -1 & 1 & -4\\ 0 & -1 & -6 & 1 \end{array}\right]\;\left[\begin{array}{c} -1\\ 1\\ -1\\ 1 \end{array}\right]=\left[\begin{array}{c} -7\\ 10\\ -5\\ 6 \end{array}\right]$$

which shows that the necessary and sufficient condition is satisfied with 0.99 confidence level

$$\overset{4}{\underset{j=1}{\sum }}w_{\text{ij}}S_{j}\geq 5,\;i=2,4\in J^{+}({\text{S}}_{1}),$$

$$\overset{4}{\underset{j=1}{\sum }}w_{\text{ij}}S_{j}\leq -5,\;i=1,3\in J^{-}({\text{S}}_{1}).$$

There are 2^4^=16 saturated states. Similar tests for the other 15 saturated states were performed. The case **S**_2_=−**S**_1_=(1−11−1) is the only other saturated equilibrium state for the network.

## 3 Robustness to noise

Robustness to noise may be assessed for (a) each saturated equilibrium expression state or (b) a specified pair of saturated equilibrium and initial expression states. In case 1, we need to *compare* how many of 2^*n*^ possible saturated initial expression states converge to each saturated equilibrium expression state; in case 2, we need to determine how many *neighbours* (differing only in one element) of the **S**(0) converge to the same saturated equilibrium expression state for a given *W*[13]. In either case, we need to establish the condition under which a saturated initial expression state converges to a given saturated equilibrium expression state of *W*.

### 3.1 Relationship between saturated initial expression states and saturated equilibrium expression states

#### 

**Theorem2**. If **S** is a saturated equilibrium expression state (or a fixed point attractor) of dynamics (3) with a given *W*, so is −**S**.

#### 

*Proof*. Since **S** is a saturated fixed point attractor of (3) with a given *W*, then (15) is satisfied. Multiplying by −1 within and outside the parentheses in (15) will not change its right-hand side:

(16) $$\begin{array}{l} (-1)S_{i}\left((-1)\overset{n}{\underset{j=1}{\sum }}w_{\text{ij}}S_{j}\right)\geq \beta ,\;(i=1,2,\ldots ,n),\\ \;(-S_{i})\left(\overset{n}{\underset{j=1}{\sum }}w_{\text{ij}}(-S_{j})\right)\geq \beta ,\;(i=1,2,\ldots ,n) \end{array}$$

which proves that −**S** is also a saturated fixed point attractor, i.e., a saturated equilibrium expression state for *W*. Example 1 demonstrates its validity. □

#### 

**Corollary1**. Dynamics (3) either does not have a saturated equilibrium expression state, or has an even number of saturated equilibrium expression states.

This result can be obtained immediately from Theorem 2.

#### 

**Example2**. Consider a gene network given by

(17) $$W=\left[\begin{array}{cccccc} \;0 & \;1 & \;1 & -1 & -1 & -1\\ \;1 & \;0 & \;1 & -1 & -1 & -1\\ \;1 & \;1 & \;0 & -1 & -1 & -1\\ -1 & -1 & -1 & \;0 & \;1 & \;1\\ -1 & -1 & -1 & \;1 & \;0 & \;1\\ -1 & -1 & -1 & \;1 & \;1 & \;0 \end{array}\right]$$

with the discrete values [-1, 0, 1] for *w*_*ij*_, and there is no auto-regulation (*w*_*ii*_=0). The six genes are separated into two groups {1,2,3} and {4,5,6}. The regulations are activating within each group, but repressing between the two groups. For such a simple system, it is easy to find by observation that amongst the 2^6^=64 saturated states only two states

$$\begin{array}{cc} {\text{S}}_{1} & =(\begin{array}{cccccc} 1 & 1 & 1 & -1 & -1 & -1 \end{array}),\\ {\text{S}}_{2} & =(\begin{array}{cccccc} -1 & -1 & -1 & 1 & 1 & 1 \end{array}) \end{array}$$

satisfy the necessary and sufficient condition (15) to be saturated equilibrium expression states with 0.99 confidence level. An examination of (15) for all 64 saturated states proved this to be the case. Moreover,

$${\text{S}}_{2}=-{\text{S}}_{1}$$

satisfies Theorem 2 and Corollary 1.

#### 

**Theorem3**. If **S**(*t*) converges to a saturated equilibrium expression state **S**, then −**S**(*t*) converges to −**S**.

#### 

*Proof*. See Theorem A3 in the Appendix. □

#### 

**Figure 3:**
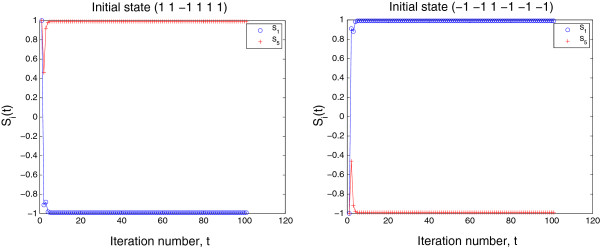
**Projections of two trajectories with initial states S**_***1***_**(*0*) and S**_***2***_**(*0*)(=−S**_***1***_**(*0*)), respectively, in Example **3** onto two-dimensional (*S***_***1***_**,*S***_***5***_**)-subspace.**

**Example3**. The gene network given in (17) is used to show the validity of Theorem 3. The following two initial saturated states

$$\begin{array}{ll} {\text{S}}_{1}(0) & =(\begin{array}{cccccc} 1 & 1 & -1 & 1 & 1 & 1 \end{array}),\\ {\text{S}}_{2}(0) & =(\begin{array}{cccccc} -1 & -1 & 1 & -1 & -1 & -1 \end{array}), \end{array}$$

with **S**_2_(0)=−**S**_1_(0), were used for dynamics (3). The two solution trajectories are found to satisfy Theorem 3. Figure 3 gives the projections of the two trajectories onto the two-dimensional (*S*_1_,*S*_5_)-subspace.

#### 

**Corollary2**. In the hypercube [−1,1]^*n*^, the volume of the region where points converge to a saturated equilibrium state **S** is equal to the volume of the region where points converge to −**S**.

#### 

*Proof*. Considering that **S**(*t*) and −**S**(*t*) are symmetric and have the same distance to the origin (see Figure 3), then in [−1,1]^*n*^ (which is symmetric to the origin) the volume of the region where points converge to **S** is equal to the volume of the region where points converge to −**S**. However, if **S**_1_ and **S**_2_ are two saturated fixed point attractors, but **S**_2_≠−**S**_1_, for a given *W*, generally in [−1,1]^*n*^ the volume of the region where points converge to **S**_1_ may not be equal to the volume of the region where points converge to **S**_2_. □

#### 

**Theorem4**. **S**=**0** is a fixed point, but may not be a fixed point attractor for a *W* having a saturated fixed point attractor **S**.

#### 

*Proof*. **S**=**0** is a fixed point because

(18) $$\begin{array}{c} \;S_{i}(t+\tau )=\frac{2}{1+\exp\left[-\overset{n}{\underset{j=1}{\sum }}w_{\text{ij}}0\right]}-1=\frac{2}{1+1}-1=0,\;\forall \mathrm{i.} \end{array}$$

Let $B_{\varepsilon }=\{\hat{S}\in {ℜ}^{n}|∥\hat{S}∥<\varepsilon \}$ be an open ball of radius *ε* centered at the origin in ℜ^*n*^ where *ε* is chosen sufficiently small such that there is only a single fixed point **0** within *B*_*ε*_. The *sufficient* condition for **0** to be a unique fixed point attractor is (see (110) of Theorem A1 in the Appendix)

(19) $$\overset{n}{\underset{j=1}{\sum }}|w_{\text{ij}}|\leq 2,\;(i=1,2,\ldots ,n).$$

A saturated fixed point attractor **S** of *W* must satisfy (15). Then, we have

(20) $$\begin{array}{ll} \;\overset{n}{\underset{j=1}{\sum }}|w_{\text{ij}}| & \geq \overset{n}{\underset{j=1}{\sum }}w_{\text{ij}}S_{i}S_{j}\\ & =S_{i}\left(\overset{n}{\underset{j=1}{\sum }}w_{\text{ij}}S_{j}\right)\geq \beta >2,\;(i=1,2,\ldots ,n). \end{array}$$

Therefore, **0** may not be a fixed point attractor, but an unstable fixed point. For an unstable fixed point, there exist *divergent* or both *convergent and divergent* neighborhoods of **0**. In the convergent region, trajectories will be attracted to **0**; In the divergent region, trajectories will leave from the neighbourhood of **0**. Therefore, it is possible upon starting from a saturated initial expression state **S**(0) that the solution trajectory arising from the sigmoidal function (3) may converge to **0**, or first approach **0**, but then enter the divergent region, and move away from that neighborhood. □

#### 

**Theorem5**. A saturated initial expression state **S**(0) converges to a saturated equilibrium expression state **S** if the following condition is satisfied after a finite number *k* of iteration steps of (3) starting from **S**(0)

(21) $$\begin{array}{c} \;\overset{n}{\underset{j=1}{\sum }}w_{\text{ij}}S_{j}(t\geq k)>\;-\;\ln\left[\;(\alpha_{i}\;-\;1)\;-\;\sqrt{{\left(\alpha_{i}\;-\;1\right)}^{2}-1}\right],\;i\in J^{+}(\text{S}), \end{array}$$

(22) $$\begin{array}{c} \;\overset{n}{\underset{j=1}{\sum }}w_{\text{ij}}S_{j}(t\geq k)<\ln\left[\;(\alpha_{i}-1)-\sqrt{{\left(\alpha_{i}-1\right)}^{2}-1}\right],\;\;\;i\in J^{-}(\text{S}), \end{array}$$

where

(23) $$\alpha_{i}=\overset{n}{\underset{j=1}{\sum }}|w_{\text{ij}}|$$

under the constraint that *α*_*i*_≥2.

#### 

*Proof*. See Theorem A4 in the Appendix. □

Theorem 5 provides a way to determine all possible saturated initial expression states converging to a saturated equilibrium expression state of a *W*. The constraint *α*_*i*_≥2 implies that the gene network must contain more than one gene if *w*_*ij*_ only takes values in [ −1,0,1]. This is always true.

#### 

**Example4**. For the gene network given in (17) all *α*_*i*_=5, and the sufficient condition for a saturated initial expression state converging to a given saturated equilibrium expression state **S** is then

(24) $$\overset{n}{\underset{j=1}{\sum }}w_{\text{ij}}S_{j}(t\geq k)>\;2.0634,\;i\in J^{+}(\text{S}),$$

(25) $$\overset{n}{\underset{j=1}{\sum }}w_{\text{ij}}S_{j}(t\geq k)<\;-2.0634,\;i\in J^{-}(\text{S}),$$

where **S**(*t*≥*k*) is the solution of (3) after *k* iterations starting from a saturated state **S**(0). All 2^6^=64 saturated states are used as initial expression states for dynamics (3). The results are given in Table 1.

**Table 1:** **Number of saturated initial states converging to different final states for the gene network given in (**17**)**

**Final state**	**Number of saturated initial states**
(1 1 1 −1 −1 −1)	22
(−1 −1 −1 1 1 1)	22
(0 0 0 0 0 0)	20

Using the condition in (24,25), we found that each saturated equilibrium expression state for the gene network given in (17) has 22 saturated initial expression states (including the saturated equilibrium expression state itself). The remaining 20 saturated initial states, which do not satisfy (24,25), converge to **0**.

For saturated initial expression states converging to one of the two saturated equilibrium expression states, the condition in (24,25) is satisfied starting from the iteration number *k* as either 0 or 1. For saturated initial expression states converging to **0**, the condition given in (24,25) is never satisfied. Therefore, in this gene network, the sigmoidal function (3) either does not need to be solved, or only needs to be solved just once, to determine which and how many saturated initial expression states converge to a particular saturated equilibrium expression state. The computational effort will be reduced. For a network with 11 genes, using Theorem 5 has approximately 60% CPU time saving compared to completely solving (3).

**Figure 4:**
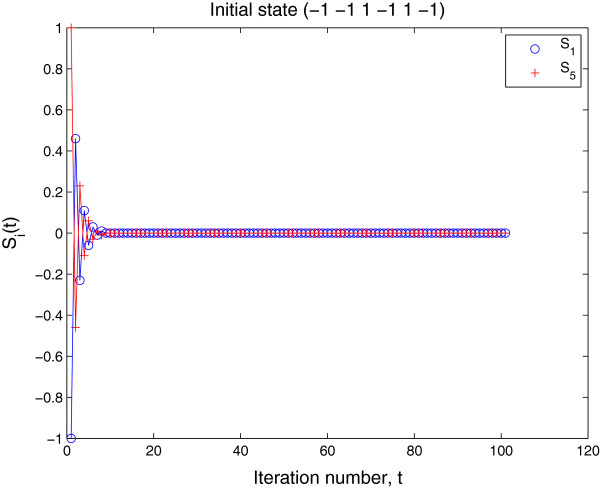
**Projection of trajectory onto two-dimensional (*S***_***1***_**,*S***_***5***_**)-subspace starting from initial state (−1 −1 1 −1 1 −1) converging to final state 0 (Example **4**).**

Figure 4 gives the projection of a trajectory converging to the final state **0** onto the two-dimensional (*S*_1_,*S*_5_)-subspace. Since **0** may be an unstable fixed point, and the values of the elements of **S**(*t*) are not exactly zero, but are within a small region around **0**. Therefore, the trajectory is sensitive to computational precision, that determines which region the trajectory enters. Continued iteration may lead to the trajectory entering the diverging region of **0** and leaving away from **0**. When there are no limit cycles for the *W*, the trajectory must converge to one of the two saturated equilibrium expression states. The outcome depends on the precision used in the computation.

**Figure 5:**
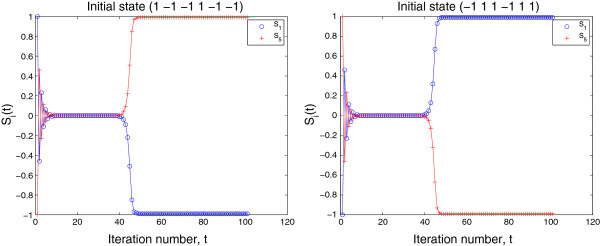
**Projections of two trajectories onto two-dimensional (*S***_***1***_**,*S***_***5***_**)-subspace (Example **4**).** The trajectories initially converge towards **0** and then leave the divergent neighborhood of **0** and converge to one of the two saturated equilibrium expression states.

Figure 5 shows two trajectories that first approach to **0** and then leave the neighborhood of **0** and converge to one of the two saturated equilibrium expression states (1 1 1 −1 −1 −1) and (−1 −1 −1 1 1 1), respectively. Such a property of **0** for dynamics (3) may not be meaningful biologically and causes confusion. This problem will be discussed in Section 5.

### 3.2 Definition for robustness to noise

Robustness to noise may be defined as follows for a network *W*, for each of its saturated equilibrium expression states, and for a specified pair of saturated equilibrium and initial expression states, respectively.

#### 

**Definition2**. The robustness to noise $R_{n_{t}}$ of a given gene network *W* is specified as inversely proportional to the total number *m* of fixed points.

(26) $$R_{n_{t}}=\left\{\begin{array}{ll} 0, & \text{if}\;\;m=0,\\ 1/m, & \text{otherwise}. \end{array}\right)$$

The subscript *n*_*t*_ denotes ‘noise’ and ‘total’.

According to the definition, larger values of *m* correspond to worse robustness to noise. This definition is reasonable because more fixed points a *W* has, then less saturated initial states converging to each of its equilibrium states. Changing an initial state has more chances to cause change of the equilibrium state.

In this definition, *m* should also include limit cycles. Since there is no simple way (like the Banach fixed point theorem for the existence of fixed point attractor) to determine the existence of limit cycle for sigmoidal functions, in the current work, we are unable to include it. The robustness $R_{n_{t}}$ takes on values in [0,1]. For *W* given in Example 2, there are three fixed points: **0**, (1 1 1 − 1 −1 −1) and (−1 −1 −1 1 1 1). Therefore, $R_{n_{t}}=1/3$, which implies that there is a 2/3 probability for having a saturated equilibrium expression state change caused by a saturated initial expression state change.

#### 

**Definition3**. The robustness to noise $R_{n_{i}}$ of a given saturated equilibrium expression state **S**_*i*_ for a gene network *W* is specified by the ratio of the number *N*_*i*_ of saturated initial expression states converging to **S**_*i*_, to the total number 2^*n*^ of possible saturated initial states

(27) $$R_{n_{i}}=N_{i}/2^{n}.$$

The subscripts *n* and *i* denote ‘noise’ and the *i*th saturated equilibrium expression state. For saturated equilibrium expression states (1 1 1 −1 −1 −1) and (−1 −1 −1 1 1 1) in Example 2, $R_{n_{i}}=22/64\approx 1/3\;(i=1,2)$.

Ciliberti et al. [13] argued that the pair of saturated equilibrium and initial expression states, **S**(*∞*) and **S**(0), play a central role for a viable network, but the variation of initial expression state in realistic cases is often mild. They define one measure of robustness to noise as the probability *R*_*v*,*I*_ that a change in *one* gene’s expression state in the saturated initial expression state **S**(0) leaves the unchanged network’s saturated equilibrium state **S**(*∞*). Following this pattern, we also define the measure of robustness to noise for a given pair of saturated equilibrium and initial expression states with a viable network as follows.

#### 

**Definition4**. The robustness to noise $R_{n_{\text{ij}}}$ of a given pair of saturated equilibrium and initial expression states **S**_*i*_ and **S**_*j*_(0) for a viable gene network *W* is specified by the ratio of the number *N*_*ij*_ of neighboring saturated initial expression states differing from **S**_*j*_(0) by only one element and still converging to **S**_*i*_, with respect to the total number *n* of possible one element differing saturated initial states

(28) $$R_{n_{\text{ij}}}=N_{\text{ij}}/\mathrm{n.}$$

#### 

**Example5**. Determination of $R_{n_{\text{ij}}}$ for the network in Example 2.

The network (17) has two equilibrium states **S**_1_, **S**_2_ with 22 saturated initial states **S**_*j*_(0) converging to each. The $R_{n_{\text{ij}}}$ for each pair of **S**_*i*_ and **S**_*j*_(0) was determined from solving dynamics (3) for all *n* one-element differing saturated initial states. The measure $R_{n_{\text{ij}}}$s for all possible pairs take only two values 1, 1/3 (i.e., *N*_*ij*_ only takes value 6 or 2). The distribution of $R_{n_{\text{ij}}}$ (i.e., how may pairs have the same $R_{n_{\text{ij}}}$) is given in Table 2.

**Table 2:** **Distribution of**$R_{n_{\mathrm{ij}}}$** for the network*W* in Example **2

**Final state**	$R_{n_{\mathrm{ij}}}$	
	**1**	**1/3**
(1 1 1 -1 -1 -1)	7	15
(-1 -1 -1 1 1 1)	7	15

For **S**_*i*_, the seven **S**_*j*_(0)s in the 22 saturated initial states with $R_{n_{\text{ij}}}=1$ are **S**_*i*_ itself and those differing from **S**_*i*_ by only one element; the other fifteen **S**_*j*_(0)s in the 22 saturated initial states with $R_{n_{\text{ij}}}=1/3$ are those differing from **S**_*i*_ by two elements. The **S**_*j*_(0) differing from **S**_*i*_ by more than two elements does not converge to the **S**_*i*_.

## 4 Robustness to mutations

For robustness to noise, we need to find all possible saturated equilibrium expression states for a given gene network *W*. In contrast, for robustness to mutations, we have the opposite task: for a given saturated equilibrium expression state we need to determine which and how many *W*s share this saturated equilibrium expression state.

### 4.1 Conditions under which gene networks share the same saturated equilibrium expression state

#### 

**Theorem6**. For a given saturated expression state **S**, all possible networks specified by particular *W*s having it as a saturated equilibrium expression state can be completely constructed by solving the following inequalities:

(29) $$\begin{array}{l} \underset{j\in J^{+}(\text{S})}{\sum }w_{\text{ij}}-\underset{j\in J^{-}(\text{S})}{\sum }w_{\text{ij}}\geq \;\beta ,\;\text{if}\;i\in J^{+}(\text{S}), \end{array}$$

(30) $$\begin{array}{l} \;\underset{j\in J^{+}(\text{S})}{\sum }w_{\text{ij}}-\underset{j\in J^{-}(\text{S})}{\sum }w_{\text{ij}}\leq -\beta ,\;\text{if}\;i\in J^{-}(\text{S}), \end{array}$$

under the condition *w*_*ij*_∈[−*a*,*a*].

#### 

*Proof*. Note that the rows of *W* are independent. When **S** is the saturated equilibrium expression state of *W*, the elements of each row (for example, the *i*th row *w*_*ij*_(*j*=1,2,…,*n*)) of *W* must satisfy either (13) or (14), i.e., (29) or (30) which is an inequality with *n* variables. The inequality is solvable and has an infinite number of solutions. Those are the desired solutions with each *w*_*ij*_ located within the required range [−*a*,*a*]. For discrete *w*_*ij*_ (only taking values [−1,0,1]), the number of solutions is finite. All the solutions can be completely counted and determined by solving (29) or (30). □

From (29) or (30), we may draw the following conclusions: 

1. For *i*∈*J*^+^(**S**), increasing the value of the first term of (29) (if the increase does not make the value of *w*_*ij*_ larger than the upper bound *a*) will not violate the inequality, i.e., increasing the value of *w*_*ij*_(*j*∈*J*^+^(**S**)) will keep the same saturated equilibrium state **S**. This behavior implies that either increasing the activation or decreasing the repression influence of active gene *j* on active gene *i* at equilibrium state **S** will not change the saturated equilibrium state.

2. For *i*∈*J*^+^(**S**), decreasing the value of the second term of (29) (if the decrease does not make the value of *w*_*ij*_ smaller than the lower bound −*a*) will not violate the inequality, i.e., decreasing the value of *w*_*ij*_(*j*∈*J*^−^(**S**)) will keep the same saturated equilibrium state **S**. This behavior implies that either decreasing the activation or increasing the repression influence of inactive gene *j* on active gene *i* at equilibrium state **S** will not change the saturated equilibrium state.

3. Similarly, for *i*∈*J*^−^(**S**), either increasing the activation or decreasing the repression influence of inactive gene *j* on inactive gene *i* at equilibrium state **S** will not change the saturated equilibrium state.

4. For *i*∈*J*^−^(**S**), either decreasing the activation or increasing the repression influence of active gene *j* on inactive gene *i* at equilibrium state **S** will not change the saturated equilibrium state.

#### 

**Example6**. Consider the determination of all possible gene networks *W* with the given saturated equilibrium expression state

$$\text{S}=(\begin{array}{llllll} 1 & 1 & 1 & -1 & -1 & -1 \end{array}).$$

Here, the discrete values [−1,0,1] are required for the elements *w*_*ij*_. Thus, we seek to determine all gene networks *W* sharing the same *two* saturated equilibrium expression states given in Example 2 (due to Theorem 2, −**S** is also a saturated equilibrium expression state).

First, consider *i*∈*J*^+^(**S**). Set *β*=5 for the 0.99 confidence level. In this case, (29) becomes

(31) $$\overset{3}{\underset{j=1}{\sum }}w_{\text{ij}}-\overset{6}{\underset{j=4}{\sum }}w_{\text{ij}}\geq 5,\;(i=1,2,3).$$

Rearrange (31) as

(32) $$\begin{array}{c} \overset{3}{\underset{j=1}{\sum }}w_{\text{ij}}\geq 5+\overset{6}{\underset{j=4}{\sum }}w_{\text{ij}}. \end{array}$$

Note that the second term on the right-hand side of (32) takes on integer values from −3 to 3 corresponding to all three *w*_*ij*_ having the value either −1 or 1, respectively. We treat each circumstance separately: 

1. $\overset{6}{\underset{j=4}{\sum }}w_{\text{ij}}=-3$

In this case, (32) is

(33) $$\begin{array}{c} \overset{3}{\underset{j=1}{\sum }}w_{\text{ij}}\geq 2. \end{array}$$

It is easy to see that there is only one choice for

$$\left(\;\begin{array}{lll} w_{i4} & w_{i5} & w_{i6} \end{array}\;)=(\;\begin{array}{lll} -1 & -1 & -1 \end{array}\;\right)$$

and four choices for

$$\;(\;\begin{array}{lll} w_{i1} & w_{i2} & w_{i3} \end{array}\;)=(\;\begin{array}{lll} 1 & 1 & 1 \end{array}\;),\;(\;\begin{array}{lll} 0 & 1 & 1 \end{array}\;),\;(\;\begin{array}{lll} 1 & 0 & 1 \end{array}\;),\;(\;\begin{array}{lll} 1 & 1 & 0 \end{array}\;).$$

Thus, we have four choices for *w*_*ij*_(*j*=1,2,…,6)

$$\begin{array}{lcr} w_{1}^{+} & = & (\;\begin{array}{llllll} 1 & 1 & 1 & -1 & -1 & -1 \end{array}\;),\\ w_{2}^{+} & = & (\;\begin{array}{llllll} 0 & 1 & 1 & -1 & -1 & -1 \end{array}\;),\\ w_{3}^{+} & = & (\;\begin{array}{llllll} 1 & 0 & 1 & -1 & -1 & -1 \end{array}\;),\\ w_{4}^{+} & = & (\;\begin{array}{llllll} 1 & 1 & 0 & -1 & -1 & -1 \end{array}\;). \end{array}$$

Here, *wk*+ denotes the *k*th permitted pattern for the *i*th row of *W* with *i*∈*J*^+^(**S**).

2. $\overset{6}{\underset{j=4}{\sum }}w_{\text{ij}}=-2$

In this case, (32) is

(34) $$\begin{array}{c} \overset{3}{\underset{j=1}{\sum }}w_{\text{ij}}\geq 3. \end{array}$$

It is easy to see that there is only one choice for

$$\left(\;\begin{array}{lll} w_{i1} & w_{i2} & w_{i3} \end{array}\;)=(\;\begin{array}{lll} 1 & 1 & 1 \end{array}\;\right)$$

and three choices for

$$\;(\;\begin{array}{lll} w_{i4} & w_{i5} & w_{i6} \end{array}\;)=(\;\begin{array}{lll} 0 & -1 & -1 \end{array}\;),\;(\;\begin{array}{lll} -1 & 0 & -1 \end{array}\;),\;(\;\begin{array}{lll} -1 & -1 & 0 \end{array}\;).$$

Thus, we have another three choices for *w*_*ij*_(*j*=1,2,…,6)

$$\begin{array}{lcr} w_{5}^{+} & = & (\;\begin{array}{llllll} 1 & 1 & 1 & 0 & -1 & -1 \end{array}\;),\\ w_{6}^{+} & = & (\;\begin{array}{llllll} 1 & 1 & 1 & -1 & 0 & -1 \end{array}\;),\\ w_{7}^{+} & = & (\;\begin{array}{llllll} 1 & 1 & 1 & -1 & -1 & 0 \end{array}\;). \end{array}$$

3. $\overset{6}{\underset{j=4}{\sum }}w_{\text{ij}}\geq -1$

In this case, (32) is

(35) $$\begin{array}{c} \overset{3}{\underset{j=1}{\sum }}w_{\text{ij}}\geq 4. \end{array}$$

This criterion is impossible because $\overset{3}{\underset{j=1}{\sum }}w_{\text{ij}}$ cannot be larger than 3. Therefore, altogether, there are only seven choices or permitted rows for *w*_*ij*_ when *i* ∈ *J*^+^(**S**).

(36) $$W^{+}=\left[\begin{array}{l} w_{1}^{+}\\ w_{2}^{+}\\ w_{3}^{+}\\ w_{4}^{+}\\ w_{5}^{+}\\ w_{6}^{+}\\ w_{7}^{+} \end{array}\right]=\left[\begin{array}{llllll} 1 & 1 & 1 & -1 & -1 & -1\\ 0 & 1 & 1 & -1 & -1 & -1\\ 1 & 0 & 1 & -1 & -1 & -1\\ 1 & 1 & 0 & -1 & -1 & -1\\ 1 & 1 & 1 & \;0 & -1 & -1\\ 1 & 1 & 1 & -1 & \;0 & -1\\ 1 & 1 & 1 & -1 & -1 & \;0 \end{array}\right].$$

Now consider *i*∈*J*^−^(**S**). In this case, (30) becomes

(37) $$\overset{3}{\underset{j=1}{\sum }}w_{\text{ij}}-\overset{6}{\underset{j=4}{\sum }}w_{\text{ij}}\leq -5,\;(i=4,5,6).$$

Rearrange (37) as

(38) $$\overset{6}{\underset{j=4}{\sum }}w_{\text{ij}}\geq 5+\overset{3}{\underset{j=1}{\sum }}w_{\text{ij}}.$$

Note that (38) is the same as (32) except that (*w*_*i*1_*w*_*i*2_*w*_*i*3_) and (*w*_*i*4_*w*_*i*5_*w*_*i*6_) interchange their positions. Therefore, there are seven choices or permitted rows for *w*_*ij*_ when *i*∈*J*^−^(**S**):

(39) $$W^{-}=\left[\begin{array}{l} w_{1}^{-}\\ w_{2}^{-}\\ w_{3}^{-}\\ w_{4}^{-}\\ w_{5}^{-}\\ w_{6}^{-}\\ w_{7}^{-} \end{array}\right]=\left[\begin{array}{llllll} -1 & -1 & -1 & 1 & 1 & 1\\ -1 & -1 & -1 & 0 & 1 & 1\\ -1 & -1 & -1 & 1 & 0 & 1\\ -1 & -1 & -1 & 1 & 1 & 0\\ \;0 & -1 & -1 & 1 & 1 & 1\\ -1 & \;0 & -1 & 1 & 1 & 1\\ -1 & -1 & \;0 & 1 & 1 & 1 \end{array}\right].$$

In the construction of *wk*+, for a given (*w*_*i*4_,*w*_*i*5_,*w*_*i*6_), all possible patterns of (*w*_*i*1_,*w*_*i*2_,*w*_*i*3_) are considered. For a given (*w*_*i*1_,*w*_*i*2_,*w*_*i*3_), a similar treatment for (*w*_*i*4_,*w*_*i*5_,*w*_*i*6_) was performed. Thus, for any *wk*+, we can always find a *wl*+ differing from it only by a single *w*_*ij*_. This is also true for *wk*−. In this example, *wk*+ and *wk*−(*k*=2,3,…,7) differ from *w*1+ and *w*1− only by a single *w*_*ij*_, respectively.

Since each row of *W* has seven choices, altogether, there are 7^6^=117,649 gene networks, each specified by a particular *W*, sharing the same saturated equilibrium expression states

$$\begin{array}{lcr} \text{S} & = & (\;\;\;\;\begin{array}{llllll} 1 & 1 & 1 & -1 & -1 & -1 \end{array}\;),\\ -\text{S} & = & (\;\begin{array}{llllll} -1 & -1 & -1 & 1 & 1 & 1 \end{array}\;). \end{array}$$

These gene networks sharing the same saturated equilibrium expression states are referred to as ‘viable’ networks (see [20]). This definition is different from that given by Ciliberti et al. [13], where viable networks were those sharing a prespecified pair of saturated initial and equilibrium expression states. The viable networks defined by sharing a pair of saturated initial and equilibrium expression states are a subset of the viable networks defined by sharing a saturated equilibrium expression state only.

The analysis here about viable networks implies that for a given saturated state, all viable networks having it as an equilibrium state must follow certain patterns, i.e., its rows must be chosen from finite permitted rows. The permitted rows for a given saturated equilibrium state have specific biological meaning and reflect the required connectivity patterns of each gene to other genes. This restriction distinguishes viable networks for a given equilibrium state from other viable networks with distinct equilibrium states as well as inviable networks.

### 4.2 Definitions of robustness to mutation

The number of viable networks in the example above 7^6^=117,649 itself is large, but the total number of possible gene networks with *n*=6 is $3^{6^{2}}\approx 1.5\times 10^{17}$. The fraction of viable gene networks in the total number of possible gene networks is 7^6^/3^36^≈7.8383×10^−13^, even smaller than that obtained previously in numerical simulations [13] for *n*=20. Based on the above analysis for viable gene networks, it seems plausible to define robustness to mutation for a given saturated equilibrium state as the ratio of the number of viable networks to the total number of possible networks for a given *n*. If so, it would appear that none of the viable gene networks is robust to mutation because the ratio is very small.

The latter inference is misleading because most of the possible gene networks have no similarity in topology with the viable gene networks having a specific saturated equilibrium state, and there is rarely a chance that a viable gene network will suddenly change to one of them. In normal circumstances, the structure perturbations due to mutation are small and the topology can only change gradually. A viable network may experience topology changes step-by-step, and in each step, only one *w*_*ij*_ changes. Ciliberti et al. [13] defined mutational robustness for a viable gene network as the fraction of its one-mutant neighbors that are also viable, and we follow the same criterion. In the following discussion, *w*_*ij*_ is restricted only to take the discrete values [−1,0,1].

We will use Example 5 as an illustration. A gene network *W* is viable if and only if its *i*th row belongs to one of the seven rows in *W*^+^ (if *i*∈*J*^+^(**S**)) or *W*^−^ (if *i*∈*J*^−^(**S**)), respectively. Since *w*_*ij*_ only takes on three values [−1,0,1], each *w*_*ij*_ may have two possible changes from its original value, and there is a total 2*n*^2^=2×6^2^=72 single *w*_*ij*_ changes (i.e., 2*n*^2^=72 one-mutant neighbors) for any *W*. Suppose that only one *w*_*ij*_ can change. From *W*^+^, we see that each element *w*1*j*+ in *w*1+ changing to 0 yields one of *wk*+(*k*=2,...,7), which is still viable. Other changes are not viable. Therefore, the total number of viable single *w*1*j*+ changes in *w*1+ is

(40) $$N_{w_{1}^{+}}^{v}=6.$$

However, for each *wkj*+ in *wk*+(*k*=2,…,7) only 0 changing to 1 (if *j*∈*J*^+^(**S**)) or −1 (if *j*∈*J*^−^(**S**)) gives *w*1+ which is still viable, and other changes are not viable. Thus, the total number of viable single *wkj*+ changes in *wk*+(*k*=2,…,7) is

(41) $$N_{w_{k}^{+}}^{v}=1,\;(k=2,\ldots ,7).$$

It can be proved that this is also true for *w*1− and *wk*−(*k*=2,…,7), i.e.,

(42) $$N_{w_{1}^{-}}^{v}=6,\;N_{w_{k}^{-}}^{v}=1,\;(k=2,\ldots ,7).$$

We then define robustness to mutation as follows:

#### 

**Definition5**. Robustness to mutation for a viable gene network *W* with a specified saturated equilibrium state **S**_*i*_ is

(43) $$R_{m_{i}}=\left\{\begin{array}{ll} 0, & \text{if}\;\text{W}\;\text{is inviable,}\\ \frac{N_{W}^{v}}{N_{W}}=\overset{n}{\underset{i=1}{\sum }}\frac{N_{w_{i}}^{v}}{2n^{2}}, & \text{if}\;\text{W}\;\text{is viable.} \end{array}\right)$$

Here the subscripts *m* and *i* in $R_{m_{i}}$ denote ‘mutation’ and the *i*th saturated equilibrium expression state; $N_{W}^{v}$ and $N_{w_{i}}^{v}$ respectively are the total numbers of viable single *w*_*ij*_ changes (which is also the number of one-mutant viable neighbors) of *W* and its *i*th row; *N*_*W*_ is the total number of possible single *w*_*ij*_ changes of *W*. For a given *W* with a specified saturated equilibrium state, its robustness to mutation can be readily calculated from $N_{w_{i}}^{v}$ of each row. In Example 5, since each row of a viable network must be one of the seven rows in *W*^+^ or *W*^−^, and $N_{w_{1}^{+}}^{v}=N_{w_{1}^{-}}^{v}=6$, $N_{w_{k}^{+}}^{v}=N_{w_{k}^{-}}^{v}=1(k=2,\ldots ,7)$, the value of $R_{m_{i}}$ depends on how many *w*1+ and *w*1− are contained in *W*.

#### 

**Example7**. Some inviable and viable networks *W*_*k*_(*k*=1,…,8) with respect to the saturated equilibrium expression state (1 1 1 −1 −1 −1) (and (−1 −1 −1 1 1 1) by Theorem 2) are given below and their $N_{W}^{v}$ and $R_{m_{i}}$ are given in Table 3.

$$\;\begin{array}{ll} W_{1}=\left[\begin{array}{llllll} \;0 & \;0 & \;1 & -1 & -1 & -1\\ \;1 & \;0 & \;1 & -1 & -1 & -1\\ \;1 & \;1 & \;0 & -1 & -1 & -1\\ -1 & -1 & -1 & \;0 & \;1 & \;1\\ -1 & -1 & -1 & \;1 & \;0 & \;1\\ -1 & -1 & -1 & \;1 & \;1 & \;0 \end{array}\right] & W_{2}=\left[\begin{array}{llllll} \;0 & \;1 & \;1 & -1 & -1 & -1\\ \;1 & \;0 & \;1 & -1 & -1 & -1\\ \;1 & \;1 & \;0 & -1 & -1 & -1\\ -1 & -1 & -1 & \;0 & \;1 & \;1\\ -1 & -1 & -1 & \;1 & \;0 & \;1\\ -1 & -1 & -1 & \;1 & \;1 & \;0 \end{array}\right]\\ W_{3}=\left[\begin{array}{llllll} \;1 & \;1 & \;1 & -1 & -1 & -1\\ \;1 & \;0 & \;1 & -1 & -1 & -1\\ \;1 & \;1 & \;0 & -1 & -1 & -1\\ -1 & -1 & -1 & \;0 & \;1 & \;1\\ -1 & -1 & -1 & \;1 & \;0 & \;1\\ -1 & -1 & -1 & \;1 & \;1 & \;0 \end{array}\right] & W_{4}=\left[\begin{array}{llllll} \;1 & \;1 & \;1 & -1 & -1 & -1\\ \;1 & \;0 & \;1 & -1 & -1 & -1\\ \;1 & \;1 & \;0 & -1 & -1 & -1\\ -1 & -1 & -1 & \;0 & \;1 & \;1\\ -1 & -1 & -1 & \;1 & \;0 & \;1\\ -1 & -1 & -1 & \;1 & \;1 & \;1 \end{array}\right]\\ W_{5}=\left[\begin{array}{llllll} \;1 & \;1 & \;1 & -1 & -1 & -1\\ \;1 & \;1 & \;1 & -1 & -1 & -1\\ \;1 & \;1 & \;0 & -1 & -1 & -1\\ -1 & -1 & -1 & \;0 & \;1 & \;1\\ -1 & -1 & -1 & \;1 & \;0 & \;1\\ -1 & -1 & -1 & \;1 & \;1 & \;1 \end{array}\right] & W_{6}=\left[\begin{array}{llllll} \;1 & \;1 & \;1 & -1 & -1 & -1\\ \;1 & \;1 & \;1 & -1 & -1 & -1\\ \;1 & \;1 & \;0 & -1 & -1 & -1\\ -1 & -1 & -1 & \;0 & \;1 & \;1\\ -1 & -1 & -1 & \;1 & \;1 & \;1\\ -1 & -1 & -1 & \;1 & \;1 & \;1 \end{array}\right]\\ W_{7}=\left[\begin{array}{llllll} \;1 & \;1 & \;1 & -1 & -1 & -1\\ \;1 & \;1 & \;1 & -1 & -1 & -1\\ \;1 & \;1 & \;1 & -1 & -1 & -1\\ -1 & -1 & -1 & \;0 & \;1 & \;1\\ -1 & -1 & -1 & \;1 & \;1 & \;1\\ -1 & -1 & -1 & \;1 & \;1 & \;1 \end{array}\right] & W_{8}=\left[\begin{array}{llllll} \;1 & \;1 & \;1 & -1 & -1 & -1\\ \;1 & \;1 & \;1 & -1 & -1 & -1\\ \;1 & \;1 & \;1 & -1 & -1 & -1\\ -1 & -1 & -1 & \;1 & \;1 & \;1\\ -1 & -1 & -1 & \;1 & \;1 & \;1\\ -1 & -1 & -1 & \;1 & \;1 & \;1 \end{array}\right] \end{array}$$

**Table 3:** **The robustness to mutation**$R_{m_{i}}$** of** ***W***_***k***_

***W***_***k***_	$N_{W}^{v}$	$R_{m_{i}}$	***W***_***k***_	$N_{W}^{v}$	$R_{m_{i}}$
*W*_1_	0	0	*W*_5_	21	21/72
*W*_2_	6	6/72	*W*_6_	26	26/72
*W*_3_	11	11/72	*W*_7_	31	31/72
*W*_4_	16	16/72	*W*_8_	36	36/72

*W*_1_ is inviable because its first row does not belong to any row of *W*^+^ or *W*^−^. The other *W*_*k*_ s are viable. As mentioned above, the value of $R_{m_{i}}$ of a viable *W* depends on how many *w*1+ and *w*1− are contained in the network. From *W*_2_ to *W*_8_, more and more *w*1+ and *w*1− are included. Thus, the corresponding $N_{W}^{v}$ and $R_{m_{i}}$ become larger and larger, and *W*_8_ is the most stable one with $N_{W}^{v}=36$ (i.e., having 36 one-mutant viable neighbors) and $R_{m_{i}}=1/2$.

For *n*=6 and the saturated equilibrium expression states (1 1 1 −1 −1 −1) and (−1 −1 −1 1 1 1), the viable gene networks can only take seven distinct values of $R_{m_{i}}$ given in Table 3 corresponding to *W* containing 0,1,…,6 of *w*1+ and *w*1−. Suppose *k* rows of a viable network are *w*1+ and *w*1−, then the value of its mutational robustness $R_{m_{i}}(k)$ is given by

(44) $$\begin{array}{ll} R_{m_{i}}(k) & =\overset{6}{\underset{i=1}{\sum }}V_{w_{i}}^{v}/(2\times 6^{2})=[6k+1(6-k)]/72\\ & =(5k+6)/72,\;(k=0,1,\ldots ,6). \end{array}$$

**Figure 6:**
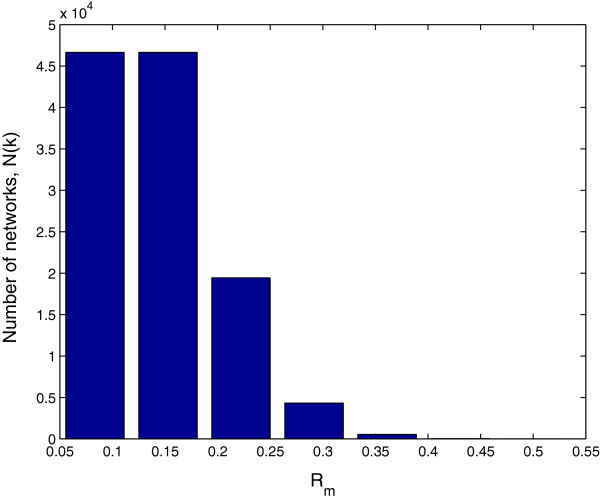
**Distribution of mutational robustness for viable gene networks.*n*** = 6; saturated equilibrium expression states (1 1 1 −1 −1 −1) and (−1 −1 −1 1 1 1).

It can be readily proved that the number *N*(*k*) of all possible viable networks with robustness to mutation equal to $R_{m_{i}}(k)$ is

(45) $$N(k)=C_{6}^{k}\times 6^{6-k},\;(k=0,1,\ldots ,6),$$

where the first term $C_{6}^{k}$ denotes the number of combinations of *k* elements taken from six elements, representing how many possible positions that *k* rows of *w*1+ or *w*1− can take in six rows of *W*. Each of the remaining 6−*k* rows of *W* has six choices from *wk*+ and *wk*−(*k*=2,…,7), and the second term 6^6−*k*^ gives the total possible number of combinations for the 6−*k* rows. Figure 6 gives the distribution of $R_{m_{i}}$.

Following [13], we also define robustness to mutation, $R_{m_{\text{ij}}}$, as follows:

#### 

**Definition6**. Robustness to mutation for a viable gene network *W* with specified saturated equilibrium and initial states **S**_*i*_ and **S**_*j*_(0) is

(46) $$R_{m_{\text{ij}}}=\frac{N_{m_{\text{ij}}}^{v}}{N_{W}}=\frac{N_{m_{\text{ij}}}^{v}}{2n^{2}}.$$

Here, the subscript *m* and *i*,*j* in $R_{m_{\text{ij}}}$ respectively denote ‘mutation’ and the *i*th saturated equilibrium expression state **S**_*i*_ and *j*th saturated initial expression state **S**_*j*_(0); $N_{m_{\text{ij}}}^{v}$ is the total numbers of viable single *w*_*ij*_ changes of *W* with respect to **S**_*i*_ and **S**_*j*_(0), which can be obtained by testing how many viable networks in $N_{W}^{v}$ share **S**_*j*_(0).

#### 

**Example8**. Determination of $R_{m_{\text{ij}}}$ for *W*_2_ and *W*_3_ in Example 7.

$R_{m_{\text{ij}}}$ values were determined for the networks *W*_2_ and *W*_3_ given in Example 7. Both *W*_2_ and *W*_3_ have the same saturated equilibrium states **S**_1_, **S**_2_. For *W*_2_ each **S**_*i*_ has 22 saturated initial states converging to it. For *W*_3_ each **S**_*i*_ has 32 saturated initial states converging to it. The numbers $N_{W}^{v}$ of one-mutant viable networks sharing the same saturated equilibrium state **S**_*i*_(*i*=1,2) for *W*_2_ and *W*_3_ are 6 and 11, respectively (see Table 3). For *W*_2_, all 6 one-mutant neighbours sharing **S**_*i*_ also share all 22 **S**_*j*_(0)(*j*=1−22), i.e., $N_{m_{\text{ij}}}^{v}=N_{W}^{v}=6$, $R_{m_{\text{ij}}}=6/72$ for all 22 pairs. For *W*_3_, $N_{m_{\text{ij}}}^{v}$ is different not only for distinct **S**_*i*_ but also for distinct initial states. For **S**_1_, $N_{m_{\text{ij}}}^{v}(\leq N_{W}^{v})$ takes values: 5,7,11; for **S**_2_, $N_{m_{\text{ij}}}^{v}(\leq N_{W}^{v})$ takes values: 5,6,9,11, respectively. Table 4 gives the distribution of $R_{m_{\text{ij}}}$ values for *W*_3_, i.e., how many pairs of **S**_*i*_ and **S**_*j*_(0) take these values.

**Table 4:** **The distribution of**$R_{m_{\mathrm{ij}}}$** for the network** ***W***_***3***_

**Final state**	$R_{m_{\mathrm{ij}}}$				
	**5/72**	**6/72**	**7/72**	**9/72**	**11/72**
**S**_**1**_	7	0	3	0	22
**S**_**2**_	1	6	0	3	22

### 4.3 Topology evolution of gene networks

From the procedure to construct viable networks given in Example 6, we know that for each permitted row there always exists another permitted row differing by only a single *w*_*ij*_ from it. Therefore, for a viable network *W*_*i*_, we can always find one or more viable networks, *W*_*j*_’s, differing by only one *w*_*ij*_ from it. The above eight networks *W*_*k*_(*k*=1,2,…,8) are an example. They only differ from one another as adjacent neighbors with a single changed *w*_*ij*_. These changes in topology correspond to the loss of a regulatory interaction (*w*_*ij*_→0), or to the appearance of a new regulatory interaction that was previously absent. The changes can be represented as a reversible path

(47) $$\begin{array}{lllllll} W_{1} & \Leftrightarrow & W_{2} & \Leftrightarrow & W_{3} & \Leftrightarrow & W_{4}\\ ↕\\ W_{8} & \Leftrightarrow & W_{7} & \Leftrightarrow & W_{6} & \Leftrightarrow & W_{5} \end{array}$$

In going from *W*_2_ to *W*_1_, the gene network no longer attains the saturated equilibrium expression state. Thus, we may consider *W*_1_ as ‘dead’. In going from *W*_2_ to *W*_3_, however, not only is the saturated equilibrium expression state retained, but also the robustness to mutation becomes higher. Suppose that all possible single *w*_*ij*_ changes have the same probability, then the gene network with higher $R_{m_{i}}$ has a greater chance to ‘survive’. This implies that highly robust topologies can evolve from topologies with low robustness through gradual Darwinian topological changes or ‘natural selection’.

Ciliberti et al. [13] suggested that all viable networks attaining a given gene expression state can be organized into a graph whose nodes are networks that differ in their topology. Two networks (nodes) in the graph are connected by an edge if they differ in the value of only one regulatory interaction (*w*_*ij*_). As proved above, for a viable network *W*_*i*_, we can always find one or more viable networks, *W*_*j*_s differing by only one *w*_*ij*_ from it. The number of viable neighbors differing by a single *w*_*ij*_ for a viable network *W* is simply the value of its $N_{W}^{v}$ (see Table 2). Therefore, any two viable networks *W*_*i*_ and *W*_*j*_ with *k* different elements *w*_*ij*_ can be connected by a path with *k* edges and *k*−1 viable networks between them. For example, *W*_2_ and *W*_8_ have six different diagonal *w*_*ij*_s, and they are connected by a path with six edges and five viable networks between them. This circumstance implies that all viable networks can be organized to comprise a large graph which can be easily traversed by a sequence of single *w*_*ij*_ changes of network topology. Thus, robustness is an evolvable property. To draw this conclusion, a previous study performed millions of simulations [13], but the analytical treatment here directly leads to this result.

## 5 Modified sigmoidal function with threshold parameters

All the results obtained here are based on the sigmoidal function model (3) for gene networks. This model is a simplified picture, and caution is called for so as to not over-interpret the conclusions obtained from our analytical treatment. For example, we proved that −**S** is also a saturated equilibrium expression state if **S** is one; this conclusion may not be biologically meaningful. Another conclusion from our analysis is that **0** may be an unstable fixed point. Following a previous definition, **0** corresponds to all genes being ‘half-on’. This definition may not be appropriate under some circumstances, and instability of **0** introduces difficulty for biological interpretation. However, these considerations provide criteria to modify the mathematical model, for example, by using the more general sigmoidal function proposed in [23] to describe network dynamics. To remove −**S** and **0** from being an equilibrium state or a fixed point, the complex sigmoidal function given in [23] is unnecessary, we only need to slightly modify the sigmoidal model (3) by introducing a threshold parameter *θ*_*i*_[21,22]:

(48) $$\begin{array}{ll} S_{i}(t+\tau ) & =\frac{2}{1+\exp\left[-\left(\overset{n}{\underset{j=1}{\sum }}w_{\text{ij}}S_{j}(t)-\theta_{i}\right)\right]}-1,\\ & \;(i=1,2,\ldots ,n). \end{array}$$

Using (48), **0** is no longer a fixed point because

(49) $$\begin{array}{ll} S_{i}(t+\tau ) & =\frac{2}{1+\exp\left[-\left(\overset{n}{\underset{j=1}{\sum }}w_{\text{ij}}0-\theta_{i}\right)\right]}-1\\ & =\frac{2}{1+e^{\theta_{i}}}-1\neq 0,\;\theta_{i}\neq 0. \end{array}$$

The necessary and sufficient condition for **S** to be an equilibrium state for (48) becomes

(50) $$\begin{array}{l} \overset{n}{\underset{j=1}{\sum }}w_{\text{ij}}S_{j}\geq \;\;\;\beta +\theta_{i},\;\;\text{if}\;\;i\in J^{+}(\text{S}), \end{array}$$

(51) $$\begin{array}{l} \overset{n}{\underset{j=1}{\sum }}w_{\text{ij}}S_{j}\leq -\beta +\theta_{i},\;\;\text{if}\;\;i\in J^{-}(\text{S}). \end{array}$$

Multiplying both sides by −1 and interchanging ≥ and ≤ and changing *J*^+^(**S**) to *J*^−^(−**S**) and *J*^−^(**S**) to *J*^+^(−**S**) yield

(52) $$\begin{array}{l} \overset{n}{\underset{j=1}{\sum }}w_{\text{ij}}(-S_{j})\leq -\beta -\theta_{i},\;\;\text{if}\;\;i\in J^{-}(-\text{S}), \end{array}$$

(53) $$\begin{array}{l} \overset{n}{\underset{j=1}{\sum }}w_{\text{ij}}(-S_{j})\geq \;\;\;\beta -\theta_{i},\;\;\text{if}\;\;i\in J^{+}(-\text{S}). \end{array}$$

If *θ*_*i*_>0 for all *i*,

(54) $$\beta -\theta_{i}<\beta +\theta_{i},$$

and it is possible that

(55) $$\overset{n}{\underset{j=1}{\sum }}w_{\text{ij}}(-S_{j})≱\beta +\theta_{i},\;\;\text{if}\;\;i\in J^{+}(-\text{S}),$$

i.e., (50) may not be satisfied, and −**S** may not be a saturated equilibrium state. If *θ*_*i*_<0 for all *i*, then

(56) $$-\beta -\theta_{i}>-\beta +\theta_{i},$$

and it is possible that

(57) $$\overset{n}{\underset{j=1}{\sum }}w_{\text{ij}}(-S_{j})≰-\beta +\theta_{i},\;\;\text{if}\;\;i\in J^{-}(-\text{S}).$$

In this case, (51) may not be satisfied, and −**S** may not be a saturated equilibrium state.

If *θ*_*i*_<0 for all *i*∈*J*^+^(**S**) and *θ*_*i*_>0 for all *i*∈*J*^−^(**S**), then both (50) and (51) may not be satisfied for −**S**, and −**S** may not be a saturated equilibrium state for *W*. In Example 2, when the model (48) is used with *θ*_*i*_=−2(*i*=1,2,3) and *θ*_*i*_=2(*i*=4,5,6), then the network given in (17) only has a single saturated equilibrium state (1 1 1 −1 −1 −1), and all saturated initial states converge to it. Thus, the problem reduces to choosing the parameter *θ*_*i*_ and giving it biological interpretation. Then, we have

### 

**Theorem7**. The necessary and sufficient condition for a saturated state **S** to be an equilibrium expression state or a fixed point attractor of the dynamics (48) with a given matrix *W* is

(58) $$\begin{array}{l} \underset{j\in J^{+}(\text{S})}{\sum }w_{\text{ij}}-\underset{j\in J^{-}(\text{S})}{\sum }w_{\text{ij}}-\theta_{i}\geq \;\;\;\beta ,\;\text{if}\;i\in J^{+}(\text{S}), \end{array}$$

(59) $$\begin{array}{l} \underset{j\in J^{+}(\text{S})}{\sum }w_{\text{ij}}-\underset{j\in J^{-}(\text{S})}{\sum }w_{\text{ij}}-\theta_{i}\leq -\beta ,\;\text{if}\;i\in J^{-}(\text{S}), \end{array}$$

or equivalently

(60) $$S_{i}\left(\overset{n}{\underset{j=1}{\sum }}w_{\text{ij}}S_{j}-\theta_{i}\right)\geq \beta ,\;(i=1,2,\ldots ,n).$$

### 

*Proof*. If a saturated state **S** is a fixed point of (48), it must satisfy (58,59), which implies that

(61) $$\lim_{t\to \infty }S_{i}(t)\approx \left\{\begin{array}{ll} \;1, & \;\text{if}\;\;S_{i}=1\\ -1, & \;\text{if}\;\;S_{i}=-1 \end{array}\right)\;(i=1,2,\ldots ,n),$$

i.e., **S** is a saturated fixed point with 0.99 (*β*=5) or 0.9999 (*β*=10) confidence level. A saturated state **S** satisfying (58,59, or 60) is a fixed point attractor. The proof is given in Theorem A5 in the Appendix. □

Similarly, we can have

### 

**Theorem8**. For a given saturated expression state **S**, all possible networks specified by particular *W*s having it as a saturated equilibrium expression state for dynamics (48) can be completely constructed by solving the following system of inequalities

(62) $$\begin{array}{l} \underset{j\in J^{+}(\text{S})}{\sum }w_{\text{ij}}-\underset{j\in J^{-}(\text{S})}{\sum }w_{\text{ij}}-\theta_{i}\geq \;\;\;\beta ,\;\text{if}\;i\in J^{+}(\text{S}), \end{array}$$

(63) $$\begin{array}{l} \underset{j\in J^{+}(\text{S})}{\sum }w_{\text{ij}}-\underset{j\in J^{-}(\text{S})}{\sum }w_{\text{ij}}-\theta_{i}\leq -\beta ,\;\text{if}\;i\in J^{-}(\text{S}), \end{array}$$

under the condition *w*_*ij*_∈[−*a*,*a*], and *θ*_*i*_∈[−*b*,*b*].

### 

*Proof*. The proof is the same as that for Theorem 6. □

## 6 Application to a yeast cell-cycle network

**Figure 7:**
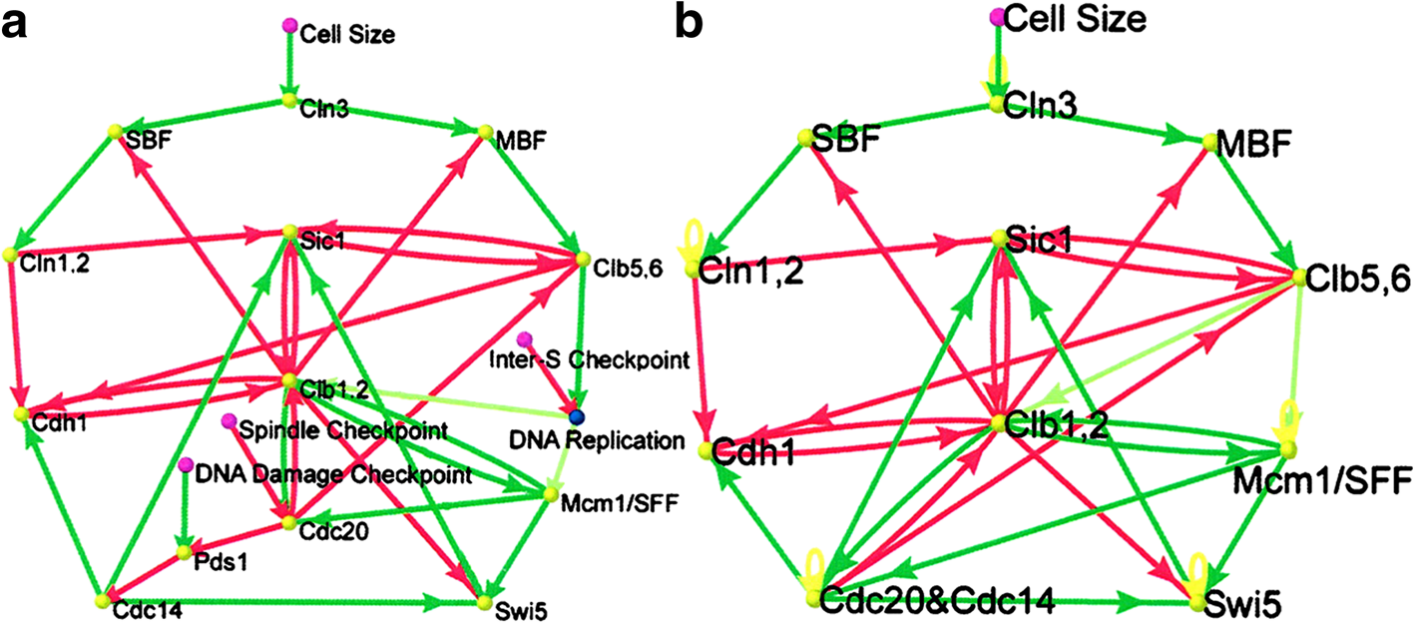
**Cell-cycle network of budding yeast (a) and its simplification with only one checkpoint ‘cell size’ (b).** Adapted from [24].

A simple yeast cell-cycle network shown in Figure 7b with 11 nodes was proposed by Li et al. [24].

The dynamics of the network was defined by Li et al. as

(64) $$S_{i}(t+1)=\left\{\begin{array}{ll} 1, & \underset{j}{\sum }a_{\text{ij}}S_{j}(t)>0,\\ 0, & \underset{j}{\sum }a_{\text{ij}}S_{j}(t)<0,\\ S_{i}(t), & \underset{j}{\sum }a_{\text{ij}}S_{j}(t)=0, \end{array}\right)$$

where 1 and 0 correspond to active and inactive states of the gene, i.e., 0 instead of −1 is used to represent the inactive state, and *a*_*ij*_ is *w*_*ij*_ in (3). Using model (64), Li et al. found that there exist 7 saturated fixed point attractors (considering 0 as −1) and all of the 2^11^=2,048 possible saturated initial expression states converge to one of the seven fixed point attractors (see Table 5).

**Table 5:** Fixed point attractors of the cell-cycle network and the number of saturated states (basin size) converging to them

**Basin**	**Cln3**	**MBF**	**SBF**	**Cln1,2**	**Cdh1**	**Swi5**	**Cdc20**	**Clb5,6**	**Sic1**	**Clb1,2**	**Mcm1**
**size**											
1,764	0	0	0	0	1	0	0	0	1	0	0
151	0	0	1	1	0	0	0	0	0	0	0
109	0	1	0	0	1	0	0	0	1	0	0
9	0	0	0	0	0	0	0	0	1	0	0
7	0	1	0	0	0	0	0	0	1	0	0
7	0	0	0	0	0	0	0	0	0	0	0
1	0	0	0	0	1	0	0	0	0	0	0

Note that dynamics (64) is different from dynamics (3). For $\underset{j}{\sum }a_{\text{ij}}S_{j}(t)=0$, dynamics (3) gives *S*_*i*_(*t*+1)=0, not *S*_*i*_(*t*). Dynamics (3) with the *W* constructed directly from the connectivities in Figure 7b will not give the same result as that given by dynamics (64). All the information given by the simplified model for yeast cell-cycle network (Figure 7b) will be considered as ‘available experimental information’ for budding yeast, and used as an example to illustrate our analytical treatment for network construction and its robustness analysis. Hereafter, 0 representing the inactive state by Li et al. will be replaced by −1. Only the main results are presented here; see the online Supplementary information (Additional file 1) for more details.

### 6.1 Construction of viable networks

Define the node order from 1 to 11 as specified in Table 5, i.e., Cln3 is node 1, MBF is node 2, etc. We first construct all viable networks sharing the most stable saturated equilibrium expression state, the first fixed point attractor in Table 5

(65) $${\text{S}}_{1}=(\;\begin{array}{lllllllllll} -1 & -1 & -1 & -1 & 1 & -1 & -1 & -1 & 1 & -1 & -1\; \end{array}\;)$$

for dynamics (3). According to Theorem 2, these viable networks will also share the other saturated equilibrium expression state

(66) $${\text{S}}_{2}=-{\text{S}}_{1}.$$

As mentioned by Li et al. [24], ‘the overall dynamic properties of the network are not very sensitive to the choice of these parameters’ (*w*_*ij*_), but the connectivity patterns of the network, i.e., the regulatory influence between genes (activation, repression, and absence) is important for determining gene network robustness. Therefore, we restrict *w*_*ij*_ to only take the discrete values 1 (activation), −1 (repression), and 0 (absence).

Theorem 6 gives the criterion to construct all of such networks *W*. When *w*_*ij*_ only takes values [−1,0,1], to satisfy (29,30) each row of *W* must have five or more nonzero elements due to *β*≥5. Otherwise, the network would not have any saturated equilibrium states. This problem occurs not only for networks with less than five genes, but also for larger networks with sparse connectivities between genes. For example, Node 1 (Cln3) in Figure 7b is a pure ‘parent’ node, which does not have any regulation coming from all other ‘children’ nodes, i.e., all *w*_1*j*_=0 for *j*≠1, and for **S**_1_ the condition (30) does not hold:

(67) $$\underset{j\in J^{+}({\text{S}}_{1})}{\sum }w_{1j}-\underset{j\in J^{-}({\text{S}}_{1})}{\sum }w_{1j}=-w_{11}≰-\mathrm{\beta .}$$

To avoid this problem, the factor *β* may be introduced such that

(68) W=βŴ,

so to satisfy condition (29,30), ${ŵ}_{\text{ij}}$ can only take values [−1,0,1] without any restriction on the number of nonzero elements in each row of . For the sake of notational simplicity, in the sequel, we still use *W* instead of , but write dynamics (3) as

(69) $$\begin{array}{lc} S_{i}(t+\tau ) & =\sigma \left(\overset{n}{\underset{j=1}{\sum }}w_{\text{ij}}S_{j}(t)\right)\\ & =\frac{2}{1+\exp\left[-\beta \overset{n}{\underset{j=1}{\sum }}w_{\text{ij}}S_{j}(t)\right]}-1. \end{array}$$

Conditions (29,30) then become

(70) $$\begin{array}{l} \underset{j\in J^{+}(\text{S})}{\sum }w_{\text{ij}}-\underset{j\in J^{-}(\text{S})}{\sum }w_{\text{ij}}\geq \;\;\;1,\;\text{if}\;i\in J^{+}(\text{S}), \end{array}$$

(71) $$\begin{array}{l} \underset{j\in J^{+}(\text{S})}{\sum }w_{\text{ij}}-\underset{j\in J^{-}(\text{S})}{\sum }w_{\text{ij}}\leq -1,\;\text{if}\;i\in J^{-}(\text{S}). \end{array}$$

For saturated equilibrium state **S**_1_, (70,71) may be rewritten as

(72) $$\begin{array}{l} \;-\overset{11}{\underset{j=1,j\neq 5,9}{\sum }}w_{\text{ij}}\geq \;\;\;1-w_{i5}-w_{i9},\;\;\;\text{if}\;i=5,9, \end{array}$$

(73) $$\begin{array}{l} \;-\overset{11}{\underset{j=1,j\neq 5,9}{\sum }}w_{\text{ij}}\leq -1-w_{i5}-w_{i9},\;\;\;\text{if}\;i\neq 5,9, \end{array}$$

or

(74) $$\begin{array}{l} \overset{11}{\underset{j=1,j\neq 5,9}{\sum }}w_{\text{ij}}\leq -1+w_{i5}+w_{i9},\;\;\;\text{if}\;i=5,9, \end{array}$$

(75) $$\begin{array}{l} \overset{11}{\underset{j=1,j\neq 5,9}{\sum }}w_{\text{ij}}\geq \;\;\;1+w_{i5}+w_{i9},\;\;\;\text{if}\;i\neq 5,9. \end{array}$$

Using the condition (74,75), all permitted row patterns sharing saturated equilibrium state **S**_1_ for dynamics (69) have been completely counted and determined (see Additional file 1: Supplementary information). Each row has **72,219** permitted patterns. Thus, the total number of viable networks sharing saturated equilibrium state **S**_1_ is

$$72,219^{11}\approx 2.7872\times 10^{53}.$$

As shown below, for the yeast cell-cycle network, the first row of *W* is restricted to be

$$(\begin{array}{lllllllllll} 1 & 0 & 0 & 0 & 0 & 0 & 0 & 0 & 0 & 0 & 0 \end{array}),$$

then the total number of viable networks for dynamics (69) is

$$72,219^{10}\approx 3.8594\times 10^{48}.$$

There are many choices of practically relevant networks.

Similarly, the dynamics with threshold parameters (48) is also modified as

(76) $$\;\begin{array}{ll} S_{i}(t+\tau ) & =\sigma \left(\overset{n}{\underset{j=1}{\sum }}w_{\text{ij}}S_{j}(t)\right)\\ & =\frac{2}{1+\exp\left[-\beta (\overset{n}{\underset{j=1}{\sum }}w_{\text{ij}}S_{j}(t)-\theta_{i})\right]}-1. \end{array}$$

The necessary and sufficient condition to have **S** as a saturated equilibrium state for dynamics (76) becomes

(77) $$\begin{array}{l} \underset{j\in J^{+}(\text{S})}{\sum }w_{\text{ij}}-\underset{j\in J^{-}(\text{S})}{\sum }w_{\text{ij}}-\theta_{i}\geq \;\;\;1,\;\text{if}\;i\in J^{+}(\text{S}), \end{array}$$

(78) $$\begin{array}{l} \underset{j\in J^{+}(\text{S})}{\sum }w_{\text{ij}}-\underset{j\in J^{-}(\text{S})}{\sum }w_{\text{ij}}-\theta_{i}\leq -1,\;\text{if}\;i\in J^{-}(\text{S}), \end{array}$$

and for **S**_1_ (77,78) become

(79) $$\begin{array}{l} \;\overset{11}{\underset{j=1,j\neq 5,9}{\sum }}w_{\text{ij}}+\theta_{i}\leq -1+w_{i5}+w_{i9},\;\;\;\text{if}\;i=5,9, \end{array}$$

(80) $$\begin{array}{l} \;\overset{11}{\underset{j=1,j\neq 5,9}{\sum }}w_{\text{ij}}+\theta_{i}\geq \;\;\;1+w_{i5}+w_{i9},\;\;\;\text{if}\;i\neq 5,9. \end{array}$$

Condition (79,80) can be used to construct all viable networks *W* for dynamics (76) with a given set of *θ*_*i*_s sharing the saturated equilibrium state **S**_1_. Since there is no unambiguous biological interpretation for the values of *θ*_*i*_, as [−1,0,1], to represent activation, repression, absence for *w*_*ij*_, we will not construct all such viable networks here.

### 6.2 Construction of yeast cell-cycle networks

According to the definition for the green and red arrows along with the yellow loop in [24], the network directly constructed from the connectivities of Figure 7b is

(81) $$\;W_{0}=\left[\begin{array}{lllllllllll} -1 & \;0 & \;0 & \;0 & \;0 & \;0 & \;0 & \;0 & \;0 & \;0 & \;0\\ \;1 & \;0 & \;0 & \;0 & \;0 & \;0 & \;0 & \;0 & \;0 & -1 & \;0\\ \;1 & \;0 & \;0 & \;0 & \;0 & \;0 & \;0 & \;0 & \;0 & -1 & \;0\\ \;0 & \;0 & \;1 & -1 & \;0 & \;0 & \;0 & \;0 & \;0 & \;0 & \;0\\ \;0 & \;0 & \;0 & -1 & \;0 & \;0 & \;1 & -1 & \;0 & -1 & \;0\\ \;0 & \;0 & \;0 & \;0 & \;0 & -1 & \;1 & \;0 & \;0 & -1 & \;1\\ \;0 & \;0 & \;0 & \;0 & \;0 & \;0 & -1 & \;0 & \;0 & \;1 & \;1\\ \;0 & \;1 & \;0 & \;0 & \;0 & \;0 & -1 & \;0 & -1 & \;0 & \;0\\ \;0 & \;0 & \;0 & -1 & \;0 & \;1 & \;1 & -1 & \;0 & -1 & \;0\\ \;0 & \;0 & \;0 & \;0 & -1 & \;0 & -1 & \;1 & -1 & \;0 & \;1\\ \;0 & \;0 & \;0 & \;0 & \;0 & \;0 & \;0 & \;1 & \;0 & \;1 & -1 \end{array}\right].$$

*W*_0_ does not satisfy condition (70,71) for any saturated state and does not have a saturated equilibrium state for dynamics (69). However, *W*_0_ will be used as the basis for the connectivities of the network to construct networks for dynamics (69) with the saturated equilibrium expression state **S**_1_. *The construction of networks reduces to satisfying condition (*74, 75*) or (*79, 80*) as much as possible consistent with experimental observation.*

Two yeast cell-cycle networks for dynamics (69)

(82) $$W_{1}=\left[\begin{array}{lllllllllll} \;1 & \;0 & \;0 & \;0 & \;0 & \;0 & \;0 & \;0 & \;0 & \;0 & \;0\\ \;1 & \;1 & \;0 & \;0 & \;0 & \;0 & \;0 & \;0 & \;0 & -1 & \;0\\ \;1 & \;0 & \;1 & \;0 & \;0 & \;0 & \;0 & \;0 & \;0 & -1 & \;0\\ \;0 & \;0 & \;1 & \;0 & \;0 & \;0 & \;0 & \;0 & \;0 & \;0 & \;0\\ \;0 & \;0 & \;0 & -1 & \;0 & \;0 & \;1 & -1 & \;0 & -1 & \;0\\ \;0 & \;0 & \;0 & \;0 & \;0 & \;0 & \;1 & \;0 & \;0 & -1 & \;1\\ \;0 & \;0 & \;0 & \;0 & \;0 & \;0 & -1 & \;0 & \;0 & \;1 & \;1\\ \;0 & \;1 & \;0 & \;0 & \;0 & \;0 & -1 & \;1 & -1 & \;0 & \;0\\ \;0 & \;0 & \;0 & -1 & \;0 & \;1 & \;1 & -1 & \;0 & -1 & \;0\\ \;0 & \;0 & \;0 & \;0 & -1 & \;0 & -1 & \;1 & -1 & \;0 & \;1\\ \;0 & \;0 & \;0 & \;0 & \;0 & \;0 & \;0 & \;1 & \;0 & \;1 & -1 \end{array}\right],$$

and

(83) $$W_{2}=\left[\begin{array}{lllllllllll} \;1 & \;0 & \;0 & \;0 & \;0 & \;0 & \;0 & \;0 & \;0 & \;0 & \;0\\ \;1 & \;1 & \;0 & \;0 & \;0 & \;0 & \;0 & \;0 & \;0 & -1 & \;0\\ \;1 & \;0 & \;1 & \;0 & \;0 & \;0 & \;0 & \;0 & \;0 & -1 & \;0\\ \;0 & \;0 & \;1 & \;0 & \;0 & \;0 & \;0 & \;0 & \;0 & \;0 & \;0\\ \;0 & \;0 & \;0 & -1 & \;0 & \;0 & \;1 & -1 & \;0 & -1 & \;0\\ \;0 & \;0 & \;0 & \;0 & \;0 & \;0 & \;1 & \;0 & \;0 & -1 & \;1\\ \;0 & \;0 & \;0 & \;0 & \;0 & \;0 & -1 & \;0 & \;0 & \;1 & \;1\\ \;0 & \;1 & \;0 & \;0 & \;0 & \;0 & -1 & \;1 & -1 & \;1 & \;0\\ \;0 & \;0 & \;0 & -1 & \;0 & \;1 & \;1 & -1 & \;0 & -1 & \;0\\ \;0 & \;0 & \;0 & \;0 & -1 & \;0 & -1 & \;1 & -1 & \;0 & \;1\\ \;0 & \;0 & \;0 & \;0 & \;0 & \;0 & \;0 & \;1 & \;0 & \;1 & -1 \end{array}\right]$$

have been obtained (the detailed procedure for their construction can be found in the Additional file 1: Supplementary information). *W*_1_ and *W*_2_ differ only for *w*_8,10_.

We can also use *W*_0_ without any change, but introduce the threshold parameters

(84) $$\Theta^{T}=(\begin{array}{lllllllllll} \theta_{1} & \theta_{2} & \theta_{3} & \theta_{4} & \theta_{5} & \theta_{6} & \theta_{7} & \theta_{8} & \theta_{9} & \theta_{10} & \theta_{11} \end{array})$$

for dynamics (76) satisfying condition (79, 80). One choice with the smallest magnitudes of *θ*_*i*_s

(85) $$\Theta^{T}=(\begin{array}{lllllllllll} 2 & 1 & 1 & 1 & 0 & 1 & 0 & 0 & 0 & 0 & 0 \end{array})$$

is obtained by using

(86) $$\begin{array}{l} \;\overset{11}{\underset{j=1,j\neq 5,9}{\sum }}w_{\text{ij}}+\theta_{i}=-1+w_{i5}+w_{i9},\;\;\;\text{if}\;i=5,9, \end{array}$$

(87) $$\begin{array}{l} \;\overset{11}{\underset{j=1,j\neq 5,9}{\sum }}w_{\text{ij}}+\theta_{i}=\;\;\;1+w_{i5}+w_{i9},\;\;\;\text{if}\;i\neq 5,9. \end{array}$$

### 6.3 Saturated equilibrium expression states for constructed networks

The saturated equilibrium expression states for a given network *W* in dynamics (69) can be determined by using the modified condition of (15) in Theorem 1

(88) $$S_{i}\left(\overset{n}{\underset{j=1}{\sum }}w_{\text{ij}}S_{j}\right)\geq 1,\;(i=1,2,\ldots ,11).$$

For *n*=11, there are 2^11^=2,048 saturated states. All of the 2,048 states were tested by condition (88) for *W*_1_ and *W*_2_, respectively, to determine which of them are saturated equilibrium states for *W*_1_ and *W*_2_. The test for 2,048 states took only **0.01** s by Matlab on a Dell Precision Workstation T3400. The saturated equilibrium expression states for a given network *W* with threshold vector *Θ* in dynamics (76) can be determined by using the condition

(89) $$S_{i}\left(\overset{n}{\underset{j=1}{\sum }}w_{\text{ij}}S_{j}-\theta_{i}\right)\geq 1,\;(i=1,2,\ldots ,11).$$

The saturated equilibrium expression states for *W*_1_,*W*_2_, and *W*_0_ with *Θ* are shown below. 

1. *W*_1_

*W*_1_ has two saturated equilibrium expression states for dynamics (69)

$$\begin{array}{lcr} {\text{S}}_{1} & = & (\;\begin{array}{lllllllllll} -1 & -1 & -1 & -1 & 1 & -1 & -1 & -1 & 1 & -1 & -1 \end{array}\;),\\ {\text{S}}_{2} & = & \left(\;\begin{array}{lllllllllll} 1 & 1 & 1 & 1 & -1 & 1 & 1 & 1 & -1 & 1 & 1 \end{array}\;\right) \end{array}$$

with

$${\text{S}}_{2}=-{\text{S}}_{1}.$$

2. *W*_2_

*W*_2_ has four saturated equilibrium expression states for dynamics (69)

$$\begin{array}{lcr} {\text{S}}_{1} & = & (\;\begin{array}{lllllllllll} -1 & -1 & -1 & -1 & 1 & -1 & -1 & -1 & 1 & -1 & -1 \end{array}\;),\\ {\text{S}}_{2} & = & (\;\begin{array}{lllllllllll} 1 & 1 & 1 & 1 & -1 & 1 & 1 & 1 & -1 & 1 & 1 \end{array}\;),\\ {\text{S}}_{3} & = & (\;\begin{array}{lllllllllll} -1 & 1 & -1 & -1 & 1 & -1 & -1 & -1 & 1 & -1 & -1 \end{array}\;),\\ {\text{S}}_{4} & = & (\;\begin{array}{lllllllllll} 1 & -1 & 1 & 1 & -1 & 1 & 1 & 1 & -1 & 1 & 1 \end{array}\;). \end{array}$$

with

$${\text{S}}_{2}=-{\text{S}}_{1},\;{\text{S}}_{4}=-{\text{S}}_{3}.$$

The **S**_1_ and **S**_3_ are just the 1st and 3rd fixed point attractors in Table 5.

3. *W*_0_ with *Θ* given in (85)

There is only a single saturated equilibrium state for dynamics (76)

$${\text{S}}_{1}=(\begin{array}{lllllllllll} -1 & -1 & -1 & -1 & 1 & -1 & -1 & -1 & 1 & -1 & -1 \end{array}).$$

### 6.4 Robustness to noise

First, the number of saturated initial expression states converging to each equilibrium expression state for *W*_1_,*W*_2_ is determined by either directly solving the dynamics (69) or using modified condition of Theorem 5

(90) $$\begin{array}{lll} & \beta \left(\overset{n}{\underset{j=1}{\sum }}w_{\text{ij}}S_{j}(t\geq k)\right)>-\ln\left[(\alpha_{i}-1)-\sqrt{{\left(\alpha_{i}-1\right)}^{2}-1}\right],\; & \;\\ & \;i\in J^{+}(\text{S}),\; \end{array}$$

(91) $$\begin{array}{lll} & \beta \left(\overset{n}{\underset{j=1}{\sum }}w_{\text{ij}}S_{j}(t\geq k)\right)<\;\;\;\ln\left[(\alpha_{i}-1)-\sqrt{{\left(\alpha_{i}-1\right)}^{2}-1}\right],\; & \;\\ & \;i\in J^{-}(\text{S}).\; \end{array}$$

For *W*_1_, the CPU times are **0.8** and **0.3** s, respectively to check all 2,048 saturated states, i.e., using Theorem 5 the CPU time is approximately 41% of that for the direct solving of the sigmoidal function. The results are given in Table 6. Note that for *W*_1_, *W*_2_, no saturated initial state converges to the unstable fixed point **0**. Therefore, in the calculation of $R_{n_{t}}$, we ignore **0** and only consider the saturated equilibrium states. The resultant robustness to noise measures $R_{n_{t}}$ and $R_{n_{i}}$ are given in Table 7. There are significant differences between $R_{n_{i}}(i=1,2,3,4)$ for *W*_2_. Obviously, the saturated equilibrium states **S**_1_,**S**_2_ are much more stable than **S**_3_,**S**_4_.

**Table 6:** The number of saturated initial states converging to different equilibrium states for different gene networks

**Final state**	**Number of saturated initial states**		
	***W***_***0***_**with***Θ*	***W***_***1***_	***W***_***2***_
**S**_**1**_	2,048	1,024	979
**S**_**2**_		1,024	979
**S**_**3**_			45
**S**_**4**_			45

**Table 7:** **The Robustness to noise**$R_{n_{t}}$** and**$R_{n_{i}}$** for different gene networks**

**Network**	$R_{n_{t}}$	$R_{n_{i}}$			
		**S**_***1***_	**S**_***2***_	**S**_***3***_	**S**_***4***_
*W*_0_ with *Θ*	1	1			
*W*_1_	1/2	1/2	1/2		
*W*_2_	1/4	0.478	0.478	0.022	0.022

The robustness to noise $R_{n_{\text{ij}}}$ for each pair of saturated equilibrium and initial expression states was calculated. The distribution of $R_{n_{\text{ij}}}$, i.e., how many pairs with the same value of $R_{n_{\text{ij}}}$, is given in Tables 8 and 9.

**Table 8:** **The distribution of**$R_{n_{\mathrm{ij}}}$** for the networks** ***W***_***0***_** with** ***Θ* and** ***W***_***1***_

**Final state**	$R_{n_{\mathrm{ij}}}$**(for** ***W***_***0***_**with** ***Θ*)**	$R_{n_{\mathrm{ij}}}$**(for** ***W***_***1***_**)**
	**11/11**	**10/11**
**S**_**1**_	2,048	1,024
**S**_**2**_		1,024

**Table 9:** **The distribution of**$R_{n_{\mathrm{ij}}}$** for the network** ***W***_***2***_

**Final state**	$R_{n_{\mathrm{ij}}}$								
	**2/11**	**3/11**	**4/11**	**5/11**	**6/11**	**7/11**	**8/11**	**9/11**	**10/11**
**S**_**1**_					1	12	29	146	791
**S**_**2**_					1	12	29	146	791
**S**_**3**_	1	6	21	7	3	7			
**S**_**4**_	1	6	21	7	3	7			

The results show that *W*_0_ with *Θ* is completely stable for any viable pair; for *W*_1_, there is one neighbour of **S**_*j*_(0) differing at the first element, which causes changes in the saturated equilibrium state **S**_*i*_; for *W*_2_, the distribution of $R_{n_{\text{ij}}}$ is divergent, and **S**_1_ and **S**_2_ are much more stable than **S**_3_ and **S**_4_.

### 6.5 Robustness to mutation

The $R_{m_{i}}$ values have been calculated for *W*_1_, *W*_2_, and *W*_0_ with the *Θ* given in (85) as shown in Table 10. Note that for **S**_1_, $R_{m_{i}}$ is almost the same for *W*_1_, *W*_2_, and *W*_0_ with *Θ*.

**Table 10:** **The robustness to mutation**$R_{m_{i}}$** of** ***W***_***0***_** with** ***Θ*,** ***W***_***1***_**, and** ***W***_***2***_

**Final state**	***W***_***0***_**with** ***Θ***		***W***_***1***_		***W***_***2***_	
	$N_{W}^{v}$	$R_{m_{i}}$	$N_{W}^{v}$	$R_{m_{i}}$	$N_{W}^{v}$	$R_{m_{\mathrm{si}}}$
**S**_**1**_	137	0.57	140	0.58	143	0.59
**S**_**2**_			140	0.58	143	0.59
**S**_**3**_					131	0.54
**S**_**4**_					131	0.54

**Figure 8:**
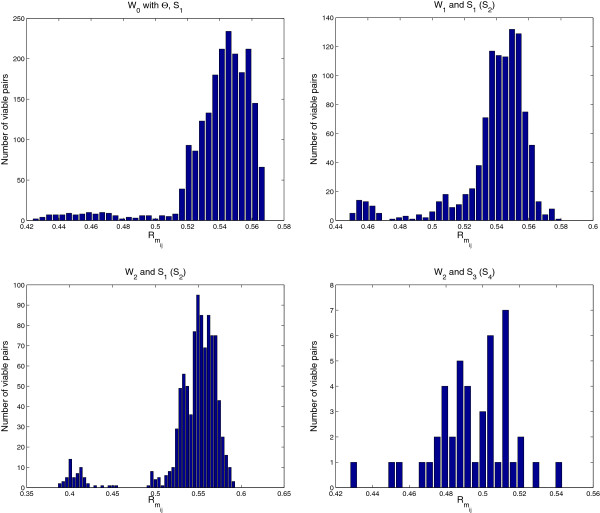
**Distribution of**$R_{m_{\mathrm{ij}}}$** for** ***W***_***1***_**,** ***W***_***2***_**, and** ***W***_***0***_** with the** ***Θ*.**

Robustness to mutation $R_{m_{\text{ij}}}$ for a viable pair of specified saturated equilibrium and initial states **S**_*i*_ and **S**_*j*_(0) has also been calculated for *W*_1_, *W*_2_, and *W*_0_ with *Θ*. The resultant distribution, i.e., how many pairs having the same $R_{m_{\text{ij}}}$ for *W*_1_, *W*_2_, and *W*_0_ with the *Θ* given above is shown in Figure 8 and Table S12 in Additional file 1: Supplementary information.

## 7 Conclusion

Based on the determination of saturated fixed point attractors for the sigmoidal function model in (3) with a given gene network, *W*, one can analytically determine which and how many saturated equilibrium expression states exist. Furthermore, for each saturated equilibrium expression state of a *W*, which and how many saturated initial expression states converging to it can also be determined. These results make it possible to establish the robustness of a given gene network to noise without performing a large number of simulations. Based on the necessary and sufficient condition for gene networks to share the same saturated equilibrium expression state, one can determine all the viable gene networks for a specified saturated equilibrium state. This result also makes it possible to establish the robustness to mutation for a network with a specified saturated equilibrium expression state or a specified pair of saturated equilibrium and initial expression states.

The analytical treatment presented here proved that for a given saturated state, all viable gene networks having it as an equilibrium state must follow certain patterns, i.e., the rows of the corresponding *W* must be chosen from a finite number of permitted rows. The permitted rows for a given saturated equilibrium state have specific biological meaning and reflect the required connectivity patterns of each gene to other genes. This restriction distinguishes the viable networks for a given saturated equilibrium state from other viable networks with distinct saturated equilibrium states as well as inviable networks. The analysis also proved, without performing a very large numbers of simulations, that all viable networks can be organized as a large graph which can be easily traversed by a sequence of single *w*_*ij*_ changes of network topology. Thus, robustness is an evolvable property. Highly robust topologies can evolve from topologies with low robustness through gradual Darwinian topological changes or natural selection. The analytical treatment presented in this paper may be employed not only for robustness analysis but also for the model construction and analysis of other properties for gene networks.

## Appendix

The appendix proves several theorems in the main text.

### **Lemma1** (**Banach Fixed Point Theorem**[25])

Let (*X*,*d*) be a non-empty complete metric space with a contraction mapping **g**:*X*→*X*. Then **g** admits a unique fixed point **x**^∗^ in *X* (i.e. **g**(**x**^∗^)=**x**^∗^). Furthermore, **x**^∗^ can be found as follows: start with an arbitrary element **x**_0_∈*X* and define a sequence {**x**_*n*_} by **x**_*n*_=**g**(**x**_*n*−1_), then **x**_*n*_→**x**^∗^.

A map **g**:*X*→*X* is called a contraction mapping on *X* if there exists *q*∈[0,1) such that

(92) d(g(x),g(y))≤qd(x,y)

where *d* denotes the distance, for all **x, y**∈*X*.

A continuous function **g** satisfies the Lipschitz condition

(93) $$∥\text{g(x)}-\text{g(y)}{∥}_{p}\leq \underset{\text{t}\in X}{\sup}∥J(\text{t}){∥}_{p}∥\text{x}-\text{y}{∥}_{p}$$

where *J*(**x**) is the Jacobian of **g(x)**. Its (*i*,*j*)th entry is

(94) $$J_{\text{ij}}(\text{x})=\left[\frac{\partial g_{i}(\text{x})}{\partial x_{j}}\right]$$

and ∥*J*(**t**)∥_*p*_ is the *L*_*p*_-norm:

(95) ∥J∥1=maxjt∈X∑i=1n∣Jij(t)∣,(j=1,2,⋯,n),

(96) ∥J∥∞=maxit∈X∑j=1n∣Jij(t)∣,(i=1,2,⋯,n),

(97) $$\begin{array}{l} \;∥J{∥}_{2}=\underset{\text{t}\in X}{\max}\sqrt{\max\lambda_{J^{T}(\text{t})J(\text{t})}}. \end{array}$$

From (95 to 97) we see that ∥*J*∥_1_ is the largest sum of the absolute values of the elements in each column; ∥*J*∥_*∞*_ is the largest sum of the absolute values of elements in each row; and ∥*J*∥_2_ is the square root of the largest eigenvalue for matrix *J*^*T*^(**t**)*J*(**t**). Now define *d* of **g(x)** and **g(y)** as the *L*_*p*_-norm of their difference. If **g** is a contraction mapping, (92) requires that at least one of the *L*_*p*_-norms of its Jacobian satisfies

(98) $$∥J{∥}_{p}\leq q<1.$$

This condition is sufficient, but not necessary. It is possible that one of (95 to 97) is satisfied, but the other two may not. Such examples can be constructed.

### 

**TheoremA1**. The sufficient condition for the sigmoidal function in (3) to have a unique fixed point attractor **0** is

(99) $$\overset{n}{\underset{j=1}{\sum }}|w_{\text{ij}}|\leq 2,\;(i=1,2,\ldots ,n).$$

### 

*Proof*. Since **0** is a fixed point for any *W*, to see whether it is a unique fixed point attractor, we need to determine under what condition (3) is a contraction mapping.

The (*i*,*j*)th entry of the Jacobian *J*^(*k*+*τ*)^(**S**(*k*)) for (3) with *τ*=1 is

(100) $$\begin{array}{ll} J_{\text{ij}}^{\left(k+1\right)}(\text{S}(k)) & =\frac{\partial S_{i}(k+1)}{\partial S_{j}(k)}=\frac{2e^{-u_{i}(k)}}{{\left(1+e^{-u_{i}(k)}\right)}^{2}}w_{\text{ij}}\\ & =\frac{2e^{-u_{i}(k)}}{1+2e^{-u_{i}(k)}+e^{-2u_{i}(k)}}w_{\text{ij}}\\ & =\frac{2}{e^{u_{i}(k)}+2+e^{-u_{i}(k)}}w_{\text{ij}} \end{array}$$

where

(101) $$u_{i}(k)=\overset{n}{\underset{j=1}{\sum }}w_{\text{ij}}S_{j}(k).$$

The matrix form of the Jacobian *J*^(*k*+1)^(**S**(*k*)) is

(102) $$J^{\left(k+1\right)}(\text{S}(k))=V(k)W$$

where *V*(*k*) is a diagonal matrix with

(103) $$V_{\text{ii}}(k)=\frac{2}{e^{u_{i}(k)}+2+e^{-u_{i}(k)}}.$$

The *∞*-norm is

(104) ∥J(k+1)(S(k))∥∞=maxiS∈[−1,1]n2eui(k)+2+e−ui(k)∑j=1n∣wij∣,(i=1,2,⋯,n).

Because $e^{u_{i}(k)},e^{-u_{i}(k)}>0$, the maximum of $2/(e^{u_{i}(k)}+2+e^{-u_{i}(k)})$ is given by the minimum of $e^{u_{i}(k)}+e^{-u_{i}(k)}$ which can be obtained from

(105) $$\frac{d\left(e^{u_{i}(k)}+e^{-u_{i}(k)}\right)}{du_{i}(k)}=e^{u_{i}(k)}-e^{-u_{i}(k)}=0.$$

This is true if and only if

(106) $$e^{u_{i}(k)}=e^{-u_{i}(k)}$$

which yields *u*_*i*_(*k*)=0, $e^{u_{i}(k)}=e^{-u_{i}(k)}=1$. The minimum for $e^{u_{i}(k)}+e^{-u_{i}(k)}$ is 2, and then

(107) maxiS∈[−1,1]n2eui(k)+2+e−ui(k)=12.

**Figure 9:**
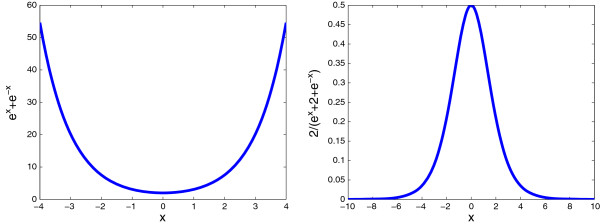
**The relations** ***e***^***x***^**+** ***e***^***−x***^** and** ***2*/(*e***^***x***^**+** ***2*** **+** ***e***^***−x***^**) with** ***x*.**

Figure 9 gives the comparison of *e*^*x*^+*e*^−*x*^ and 2/(*e*^*x*^+2+*e*^−*x*^).

Therefore, we have

(108) $$\begin{array}{c} \;∥J^{\left(k+1\right)}(\text{S}(k)){∥}_{\infty }=\underset{i}{\max}\frac{1}{2}\overset{n}{\underset{j=1}{\sum }}|w_{\text{ij}}|,\;(i=1,2,\cdots \;,n). \end{array}$$

To be a contraction mapping requires

(109) $$∥J^{\left(k+1\right)}(\text{S}(k)){∥}_{\infty }<1$$

i.e.,

(110) $$\overset{n}{\underset{j=1}{\sum }}|w_{\text{ij}}|<2,\;(i=1,2,\cdots \;,n).$$

Notice that 1/2 is the superior value in (107). In many cases, not all *S*_*i*_=0 (i.e., *u*_*i*_(*k*)’s are not zero), then the factor in (107) has values smaller than 1/2, which implies that the condition for a contraction mapping given in (110) may be softened as

(111) $$\overset{n}{\underset{j=1}{\sum }}|w_{\text{ij}}|\leq 2,\;(i=1,2,\cdots \;,n).$$

The condition in (111) is sufficient, but not necessary. □

### 

**TheoremA2**. A saturated state **S** satisfying (13) and (14) is a fixed point attractor.

### 

*Proof*. Suppose **S** is a fixed point of (3). Let $B_{\varepsilon }=\{\hat{S}\in {ℜ}^{n}|∥\text{X}=\hat{S}-\text{S}∥<\varepsilon \}$, where *ε* is chosen sufficiently small such that there is only a single fixed point **S** within *B*_*ε*_. **X**=**0** is a fixed point in *B*_*ε*_ with representation **X** because **S** is a fixed point for $\hat{S}$ in *B*_*ε*_. If we can prove that **X**=**0** is a fixed point attractor in *B*_*ε*_, then so is **S**.

Subtracting *S*_*i*_ on the both sides of (3) yields

(112) $$\begin{array}{lcr} X_{i}(t+\tau ) & = & S_{i}(t+\tau )-1=\frac{2}{1+\exp\left[-\overset{n}{\underset{j=1}{\sum }}w_{\text{ij}}S_{j}(t)\right]}-1-1\\ & = & \frac{2}{1+\exp\left[-\overset{n}{\underset{j=1}{\sum }}w_{\text{ij}}\left(S_{j}(t)-1+1\right)\right]}-2\\ & = & \frac{2}{1+\exp\left[-\overset{n}{\underset{j=1}{\sum }}\left(w_{\text{ij}}X_{j}(t)+w_{\text{ij}}S_{j}\right)\right]}-2\\ & = & \frac{2}{1+e^{-\beta^{\prime }}\exp\left[-\overset{n}{\underset{j=1}{\sum }}w_{\text{ij}}X_{j}(t)\right]}-2,\;i\in J^{+}(\text{S}) \end{array}$$

where *β*^′^≥*β*.

The (*i*,*j*)th entry of the Jacobian is

(113) $$\begin{array}{lcr} J_{\text{ij}}^{\left(t+\tau \right)}(\text{X}(t)) & = & \frac{2e^{-\beta^{\prime }}\exp\left[-\overset{n}{\underset{j=1}{\sum }}w_{\text{ij}}X_{j}(t)\right]}{{\left(1+e^{-\beta^{\prime }}\exp\left[-\overset{n}{\underset{j=1}{\sum }}w_{\text{ij}}X_{j}(t)\right]\right)}^{2}}w_{\text{ij}}\\ & = & \frac{2e^{-\beta^{\prime }}\exp\left[-\overset{n}{\underset{j=1}{\sum }}w_{\text{ij}}X_{j}(t)\right]}{1+2e^{-\beta^{\prime }}\exp\left[-\overset{n}{\underset{j=1}{\sum }}w_{\text{ij}}X_{j}(t)\right]+e^{-2\beta^{\prime }}\exp\left[-2\overset{n}{\underset{j=1}{\sum }}w_{\text{ij}}X_{j}(t)\right]}w_{\text{ij}}\\ & = & \frac{2}{e^{\beta^{\prime }}\exp\left[\overset{n}{\underset{j=1}{\sum }}w_{\text{ij}}X_{j}(t)\right]+2+e^{-\beta^{\prime }}\exp\left[-\overset{n}{\underset{j=1}{\sum }}w_{\text{ij}}X_{j}(t)\right]}w_{\text{ij}}\\ & \approx & \frac{2}{e^{\beta^{\prime }}+2+e^{-\beta^{\prime }}}w_{\text{ij}},\;i\in J^{+}(\text{S}). \end{array}$$

Here, the condition **X**(*t*)<*ε*≈0 and $\exp\left[\overset{n}{\underset{j=1}{\sum }}w_{\text{ij}}X_{j}(t)\right]\approx \exp\left[-\overset{n}{\underset{j=1}{\sum }}w_{\text{ij}}X_{j}(t)\right]\approx 1$ were used. The *L*_1_-norm of row vector $J_{i}^{\left(t+\tau \right)}$ is

(114) $$∥J_{i}^{\left(t+\tau \right)}(\text{X}(t)){∥}_{1}\approx \frac{2}{e^{\beta^{\prime }}+2+e^{-\beta^{\prime }}}\overset{n}{\underset{j=1}{\sum }}|w_{\text{ij}}|.$$

When

(115) $$\begin{array}{lcr} \overset{n}{\underset{j=1}{\sum }}|w_{\text{ij}}| & < & \frac{e^{\beta^{\prime }}+2+e^{-\beta^{\prime }}}{2}\\ & \geq & \frac{e^{\beta }+2+e^{-\beta }}{2}>\left\{\begin{array}{ll} 75, & \text{if}\;\beta =5\text{,}\\ 11,014, & \text{if}\;\beta =10\text{,} \end{array}\right) \end{array}$$

which is often the case in practice for *W* satisfying (13) and (14), we have

(116) $$∥J_{i}^{\left(t+\tau \right)}(\text{X}(t)){∥}_{1}<1,\;i\in J^{+}(\text{S}).$$

The same result can be obtained for *i*∈*J*^−^(**S**). Therefore, we have

(117) $$∥J^{\left(t+\tau \right)}(\text{X}(t)){∥}_{\infty }=\underset{i}{\max}∥J_{i}^{\left(t+\tau \right)}(\text{X}(t)){∥}_{1}<1,$$

i.e., **X**=**0** is a fixed point attractor, and thus so is **S**. □

### 

**TheoremA3**. If an **S**(*t*) converges to a saturated equilibrium expression state **S**, then −**S**(*t*) converges to the saturated equilibrium expression state −**S**.

### 

*Proof*. For **S**(*t*) and −**S**(*t*), the corresponding equations are

(118) $$S_{i}(t+\tau )=\frac{2}{1+\exp\left[-\overset{n}{\underset{j=1}{\sum }}w_{\text{ij}}S_{j}(t)\right]}-1,\;(i=1,2,\ldots ,n),$$

and

(119) $${\tilde{S}}_{i}(t+\tau )=\frac{2}{1+\exp\left[-\overset{n}{\underset{j=1}{\sum }}w_{\text{ij}}(-S_{j}(t))\right]}-1,\;(i=1,2,\ldots ,n),$$

respectively. It can be proved that

(120) $${\tilde{S}}_{i}(t+\tau )=-S_{i}(t+\tau ).$$

The proof is given below.

(121) $$\begin{array}{lcr} & & {\tilde{S}}_{i}(t+\tau )=\frac{2}{1+\exp\left[-\overset{n}{\underset{j=1}{\sum }}w_{\text{ij}}(-S_{j}(t))\right]}-1=\frac{2}{1+\exp\left[\overset{n}{\underset{j=1}{\sum }}w_{\text{ij}}S_{j}(t)\right]}-1\\ & = & \frac{1-\exp\left[\overset{n}{\underset{j=1}{\sum }}w_{\text{ij}}S_{j}(t)\right]}{1+\exp\left[\overset{n}{\underset{j=1}{\sum }}w_{\text{ij}}S_{j}(t)\right]}=\frac{1+\exp\left[\overset{n}{\underset{j=1}{\sum }}w_{\text{ij}}S_{j}(t)\right]-2\exp\left[\overset{n}{\underset{j=1}{\sum }}w_{\text{ij}}S_{j}(t)\right]}{1+\exp\left[\overset{n}{\underset{j=1}{\sum }}w_{\text{ij}}S_{j}(t)\right]}\\ & = & \frac{\left(1+\exp\left[\overset{n}{\underset{j=1}{\sum }}w_{\text{ij}}S_{j}(t)\right]-2\exp\left[\overset{n}{\underset{j=1}{\sum }}w_{\text{ij}}S_{j}(t)\right]\right)\left(1+\exp\left[-\overset{n}{\underset{j=1}{\sum }}w_{\text{ij}}S_{j}(t)\right]\right)}{\left(1+\exp\left[\overset{n}{\underset{j=1}{\sum }}w_{\text{ij}}S_{j}(t)\right]\right)\left(1+\exp\left[-\overset{n}{\underset{j=1}{\sum }}w_{\text{ij}}S_{j}(t)\right]\right)}\\ & = & 1-\frac{2\left(\exp\left[\overset{n}{\underset{j=1}{\sum }}w_{\text{ij}}S_{j}(t)\right]+1\right)}{\left(1+\exp\left[\overset{n}{\underset{j=1}{\sum }}w_{\text{ij}}S_{j}(t)\right]\right)\left(1+\exp\left[-\overset{n}{\underset{j=1}{\sum }}w_{\text{ij}}S_{j}(t)\right]\right)}\\ & = & 1-\frac{2}{1+\exp\left[-\overset{n}{\underset{j=1}{\sum }}w_{\text{ij}}S_{j}(t)\right]}=-S_{i}(t+\tau ). \end{array}$$

Equation (121) implies that there is an one-to-one relation between ${\tilde{S}}_{i}(t)$ and −*S*_*i*_(*t*). Since **S**(*∞*)=**S**, we have

(122) $$\tilde{S}(\infty )=-\text{S}.$$

Therefore, starting from −**S**(*t*) will converge to −**S**. □

### 

**TheoremA4**. A saturated initial expression state **S**(0) converges to a saturated equilibrium expression state **S** if the following condition is satisfied after a finite number *k* of iteration steps of (3) starting from **S**(0)

(123) $$\begin{array}{lll} & \overset{n}{\underset{j=1}{\sum }}w_{\text{ij}}S_{j}(t\geq k)>-\ln[(\alpha_{i}-1)-\sqrt{{\left(\alpha_{i}-1\right)}^{2}-1}],\; & \;\\ & \;\;\;\;i\in J^{+}(\text{S}),\; \end{array}$$

(124) $$\begin{array}{lll} & \overset{n}{\underset{j=1}{\sum }}w_{\text{ij}}S_{j}(t\geq k)<\ln[(\alpha_{i}-1)-\sqrt{{\left(\alpha_{i}-1\right)}^{2}-1}],\; & \;\\ & \;\;\;\;i\in J^{-}(\text{S}),\; \end{array}$$

where

(125) $$\alpha_{i}=\overset{n}{\underset{j=1}{\sum }}|w_{\text{ij}}|$$

under the constraint that *α*_*i*_≥2.

### 

*Proof*. First, consider *i*∈*J*^+^(**S**). If **S** is the fixed point attractor for the initial state **S**(**0**), then **X**=**0** is the fixed point attractor for the initial expression state **X**(0)=**S**(0)−**S** of (112). This is equivalent to finding the condition under which (112) is a contraction mapping. According to the Banach fixed point theorem, the sufficient condition is that the norm of the Jacobian matrix satisfies ∥*J*∥_*∞*_<1. To prove Theorem A4, we try to seek the largest *B*_*a*_={**X**∈ℜ^*n*^∣∥**X**∥<*a*} where *a* is the upper bound to have ∥*J*∥_*∞*_<1, i.e., $∥J_{i}^{\left(t+\tau \right)}(\text{X}(t)){∥}_{1}<1$, for all *i*∈*J*^+^(**S**).

Set

(126) $$y=e^{-\beta^{\prime }}\exp\left[-\overset{n}{\underset{j=1}{\sum }}w_{\text{ij}}X_{j}(t)\right].$$

From (113) the *L*_1_-norm of row vector $J_{i}^{\left(t+\tau \right)}$ can be represented as

(127) $$\begin{array}{lcr} \;∥J_{i}^{\left(t+\tau \right)}(\text{X}(t)){∥}_{1} & = & \frac{2y}{{\left(1+y\right)}^{2}}\overset{n}{\underset{j=1}{\sum }}|w_{\text{ij}}|\\ & = & \frac{2\alpha_{i}y}{{\left(1+y\right)}^{2}}<1,\;i\in J^{+}(\text{S}), \end{array}$$

which gives the quadratic equation

(128) $$y^{2}+2(1-\alpha_{i})y+1=(y-y_{1})(y-y_{2})>0,$$

where

(129) $$\begin{array}{l} y_{1}=(\alpha_{i}-1)+\sqrt{{\left(\alpha_{i}-1\right)}^{2}-1}, \end{array}$$

(130) $$\begin{array}{l} y_{2}=(\alpha_{i}-1)-\sqrt{{\left(\alpha_{i}-1\right)}^{2}-1}. \end{array}$$

It is easy to check that *y*_1_,*y*_2_ are all nonnegative when *α*_*i*_≥2, and *y*_2_<1.

Equation (128) is valid if and only if *y* is chosen within the two disjoint ranges

$$\begin{array}{lll} \left(-\infty ,y_{2}\right) & \left(y_{1},\infty \right) & \text{if}\;\alpha_{i}>2,\\ \left(-\infty ,\;\;1\right) & \left(1,\;\;\infty \right) & \text{if}\;\alpha_{i}=2, \end{array}$$

i.e., either smaller than *y*_2_ or larger than *y*_1_. This implies that

(131) $$\begin{array}{c} \;e^{-\beta^{\prime }}\exp\left[-\overset{n}{\underset{j=1}{\sum }}w_{\text{ij}}X_{j}(t)\right]=\exp\left[-\overset{n}{\underset{j=1}{\sum }}w_{\text{ij}}S_{j}(t)\right]\left\{\begin{array}{l} >y_{1}\\ <y_{2} \end{array}\right) \end{array}$$

or

(132) $$\overset{n}{\underset{j=1}{\sum }}w_{\text{ij}}S_{j}(t)\left\{\begin{array}{l} <-\ln[(\alpha_{i}-1)+\sqrt{{\left(\alpha_{i}-1\right)}^{2}-1}]<0,\\ >-\ln[(\alpha_{i}-1)-\sqrt{{\left(\alpha_{i}-1\right)}^{2}-1}]>0. \end{array}\right)$$

Using (15), we know that when **S**(*t*) is close to the fixed point attractor **S**,

(133) $$\overset{n}{\underset{j=1}{\sum }}w_{\text{ij}}S_{j}>0,\;i\in J^{+}(\text{S}).$$

Therefore, we obtain

(134) $$\;\overset{n}{\underset{j=1}{\sum }}w_{\text{ij}}S_{j}(t)>-\ln[(\alpha_{i}-1)-\sqrt{{\left(\alpha_{i}-1\right)}^{2}-1}].$$

If **S**(*t*=*k*) satisfies (134), then the trajectory starting from **S**(*t*=*k*) will converge to **S**. Therefore, **S**(*t*≥*k*) will also satisfy (134). Thus,

(135) $$\overset{n}{\underset{j=1}{\sum }}w_{\text{ij}}S_{j}(t\geq k)>-\ln[(\alpha_{i}-1)-\sqrt{{\left(\alpha_{i}-1\right)}^{2}-1}].$$

Equation (135) proves the condition for *i*∈*J*^+^(**S**) given in Theorem A4.

For *i*∈*J*^−^(**S**), (112) becomes

(136) $$\begin{array}{ll} \;X_{i}(t+\tau ) & =S_{i}(t+\tau )-(-1)\\ & =\frac{2}{1+e^{\beta^{\prime }}\exp\left[-\overset{n}{\underset{j=1}{\sum }}w_{\text{ij}}X_{j}(t)\right]},\;i\in J^{-}(\text{S}). \end{array}$$

Set

(137) $$y=e^{\beta^{\prime }}\exp\left[-\overset{n}{\underset{j=1}{\sum }}w_{\text{ij}}X_{j}(t)\right].$$

Then the proof procedure is the same as above except that for *i*∈*J*^−^(**S**)

(138) $$\overset{n}{\underset{j=1}{\sum }}w_{\text{ij}}S_{j}<0,\;i\in J^{-}(\text{S})$$

when **S**(*t*) is close to the fixed point attractor **S**. Thus, we obtain

(139) $$\overset{n}{\underset{j=1}{\sum }}w_{\text{ij}}S_{j}(t)<-\ln[(\alpha_{i}-1)+\sqrt{{\left(\alpha_{i}-1\right)}^{2}-1}].$$

Note that

(140) $$\;\begin{array}{l} -\ln[(\alpha_{i}-1)+\sqrt{{\left(\alpha_{i}-1\right)}^{2}-1}]\\ =\ln\frac{1}{(\alpha_{i}-1)+\sqrt{{\left(\alpha_{i}-1\right)}^{2}-1}}\\ =\ln\frac{(\alpha_{i}-1)-\sqrt{{\left(\alpha_{i}-1\right)}^{2}-1}}{\left[\;(\alpha_{i}-1\;)+\sqrt{\;{\left(\alpha_{i}-1\;\right)}^{2}-1}\;][\;(\alpha_{i}-1\;)\;-\;\sqrt{{\left(\alpha_{i}-1\;\right)}^{2}-1}\;\right]}\\ =\ln\frac{(\alpha_{i}-1)-\sqrt{{\left(\alpha_{i}-1\right)}^{2}-1}}{{\left(\alpha_{i}-1\right)}^{2}-{\left(\alpha_{i}-1\right)}^{2}+1}\\ =\ln[(\alpha_{i}-1)-\sqrt{{\left(\alpha_{i}-1\right)}^{2}-1}]. \end{array}$$

Then we have

(141) $$\overset{n}{\underset{j=1}{\sum }}w_{\text{ij}}S_{j}(t)<\ln[(\alpha_{i}-1)-\sqrt{{\left(\alpha_{i}-1\right)}^{2}-1}],$$

and similarly we obtain

(142) $$\overset{n}{\underset{j=1}{\sum }}w_{\text{ij}}S_{j}(t\geq k)<\ln[(\alpha_{i}-1)-\sqrt{{\left(\alpha_{i}-1\right)}^{2}-1}],$$

i.e., the condition for *i*∈*J*^−^(**S**) given in Theorem A4. □

### 

**TheoremA5**. A saturated state **S** satisfying (58) and (59) is a fixed point attractor.

### 

*Proof*. Suppose **S** is a fixed point of (48). Let $B_{\varepsilon }=\{\hat{S}\in {ℜ}^{n}|∥\text{X}=\hat{S}-\text{S}∥<\varepsilon \}$ where *ε* is chosen sufficiently small such that there is only a single fixed point **S** within *B*_*ε*_. **X**=**0** is a fixed point in *B*_*ε*_ with representation **X** because **S** is a fixed point for $\hat{S}$ in *B*_*ε*_. If we can prove that **X**=**0** is a fixed point attractor in *B*_*ε*_, then so is **S**.

Subtracting *S*_*i*_ on the both sides of (48) yields

(143) $$\begin{array}{l} \;X_{i}(t+\tau )=S_{i}(t+\tau )-1\\ =\frac{2}{1+\exp\left[-\left(\overset{n}{\underset{j=1}{\sum }}w_{\text{ij}}S_{j}(t)-\theta_{i}\right)\right]}-1-1\\ =\frac{2}{1+\exp\left[-\left(\overset{n}{\underset{j=1}{\sum }}w_{\text{ij}}(S_{j}(t)-1+1)-\theta_{i}\right)\right]}-2\\ =\frac{2}{1+\exp\left[-\left(\overset{n}{\underset{j=1}{\sum }}(w_{\text{ij}}X_{j}(t)+w_{\text{ij}}S_{j})-\theta_{i}\right)\right]}-2\\ =\frac{2}{1+e^{-\beta^{\prime }}\exp\left[-\overset{n}{\underset{j=1}{\sum }}w_{\text{ij}}X_{j}(t)\right]}-2,\;i\in J^{+}(\text{S}) \end{array}$$

where

(144) $$\beta^{\prime }=\overset{n}{\underset{j=1}{\sum }}w_{\text{ij}}S_{j}-\theta_{i}\geq \mathrm{\beta .}$$

The following step of proof is exactly the same as that in Theorem A2, and will not repeat here. □

## Competing interests

The authors declare that they have no competing interests.

## Supplementary Material

Additional file 1 **Supplementary information.** Application to the yeast cell-cycle network. Click here for file
