# Supplementary material for: Analysis of gene network robustness based on saturated fixed point attractors
Source: EURASIP J Bioinform Syst Biol. 2014 Mar 20;2014(1):4. doi: 10.1186/1687-4153-2014-4 (PMC3998189; doi:10.1186/1687-4153-2014-4)
Supplement: Additional file 1 — Supplementary information. Application to the yeast cell-cycle network. [file 1687-4153-2014-4-S1.pdf]

Supplemental information: Application to the yeast cell-cycle network

A simple yeast cell-cycle network shown in Figure 1(B) with 11 nodes was proposed (F. Li, et al., The yeast cell-cycle network is robustly designed. PNAS, **101**(14), 4781-4786(2004)).

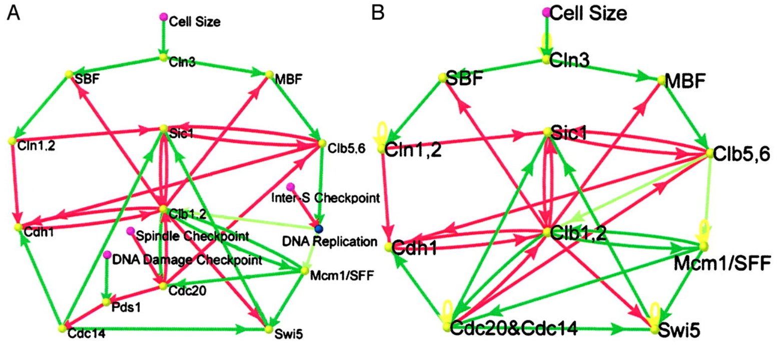

Figure 1: The cell-cycle network of the budding yeast (A) and simplified cell-cycle network with only one checkpoint "cell size" (B) (reproduced from the above paper).

The dynamics of the network was defined by Li et al. as

$$S_i(t+1) = \begin{cases} 1, & \sum_j a_{ij} S_j(t) > 0, \\ 0, & \sum_j a_{ij} S_j(t) < 0, \\ S_i(t), & \sum_j a_{ij} S_j(t) = 0, \end{cases} \tag{1}$$

where 1 and 0 correspond to active and inactive states of the gene, i.e., 0 instead of -1 is used to represent inactive state, and a_{ij} is similar to w_{ij} in dynamics

$$
\begin{aligned}
S_i(t+\tau) &= \sigma(\sum_{j=1}^{n} w_{ij} S_j(t)) \\
&= \frac{2}{1+\exp[-\sum_{j=1}^{n} w_{ij} S_j(t)]} - 1, \quad (i = 1, 2, \ldots, n),
\end{aligned} \tag{2}
$$

a_{ij} takes values 1 (green arrow) and -1 (red arrow), respectively. Moreover, the yellow loop is used to represent "self-degeneration" (value -1).

Using model (1) Li, et al. found that there exit 7 saturated fixed point attractors (upon considering 0 as -1) and all of the $2^{11} = 2,048$ possible saturated initial expression states converge to one of the seven fixed point attractors (see Table 1).

Table 1. The fixed point attractors of the yeast cell-cycle network (Figure 1(B)) and the number of saturated initial expression states (basin size) converging to them

Basin size	Cln3	MBF	SBF	Cln1,2	Cdh1	Swi5	Cdc20	Clb5,6	Sic1	Clb1,2	Mcm1
1,764	0	0	0	0	1	0	0	0	1	0	0
151	0	0	1	1	0	0	0	0	0	0	0
109	0	1	0	0	1	0	0	0	1	0	0
9	0	0	0	0	0	0	0	0	1	0	0
7	0	1	0	0	0	0	0	0	1	0	0
7	0	0	0	0	0	0	0	0	0	0	0
1	0	0	0	0	1	0	0	0	0	0	0

Note that dynamics (1) is different from dynamics (2). For $\sum_j a_{ij} S_j(t) = 0$, dynamics (2) gives $S_i(t+1) = 0$, not $S_i(t)$. Dynamics (2) with the W constructed directly from the connectivities in Figure 1(B) will not give the same result as that from dynamics (1). Hereafter, 0 representing an inactive state by Li et al. will be replaced by -1.

All the information given by the simplified model for the yeast cell-cycle network (Figure 1(B)) will be considered as "experimental information" for the budding yeast. The procedure shown below is only an illustration for network construction and robustness analysis by our analytical treatment combining the "experimental information" of budding yeast. A biologist familiar with the yeast cell-cycle network possibly may construct better networks W.

1 Network construction

Define the node order from 1 to 11 as that given in Table 1, i.e., Cln3 is node 1, MBF is node 2, etc. We first construct all viable networks sharing the most stable saturated equilibrium expression state, the first fixed point attractor in Table 1

$$\mathbf{S}_1 = (\ -1\ \ -1\ \ -1\ \ -1\ \ \ 1\ \ -1\ \ -1\ \ -1\ \ \ 1\ \ -1\ \ -1\ ) \tag{3}$$

for dynamics (2). According to Theorem 2, these viable networks will also share the other saturated equilibrium expression state

$$\mathbf{S}_2 = -\mathbf{S}_1. \tag{4}$$

Theorem 6 gives the criterion to construct all of such networks W, i.e., for the given saturated equilibrium state $\mathbf{S}_1$ the following inequalities must be satisfied by W:

$$\sum_{j\in J^+(\mathbf{S}_1)} w_{ij} - \sum_{j\in J^-(\mathbf{S}_1)} w_{ij} \geq \ \ \beta, \quad \text{if } i \in J^+(\mathbf{S}_1), \tag{5}$$

$$\sum_{j\in J^+(\mathbf{S}_1)} w_{ij} - \sum_{j\in J^-(\mathbf{S}_1)} w_{ij} \leq -\beta, \quad \text{if } i \in J^-(\mathbf{S}_1), \tag{6}$$

under the condition $w_{ij} \in [-a, a]$. Here,

$$J^+(\mathbf{S}_1) = \{5, 9\}, \tag{7}$$

$$J^-(\mathbf{S}_1) = \{1, 2, 3, 4, 6, 7, 8, 10, 11\}. \tag{8}$$

When w_{ij} can take any value within $[-a, a]$, there is an infinite number of solutions for (5,6). As mentioned by Li, et al., "the overall dynamic properties of the network are not very sensitive to the choice of these parameters" (w_{ij}), but the connectivity patterns of the network, i.e., the regulatory influence between genes (activation, repression and absence) is important for gene network robustness analysis. Therefore, we restrict that w_{ij} can only take the discrete values 1 (activation), -1 (repression) and 0 (absence).

When w_{ij} only takes values $[-1, 0, 1]$, to satisfy (5,6), then each row of W must have 5 or more nonzero elements due to $\beta \geq 5$. Otherwise, the network would not have a saturated equilibrium state. This problem occurs not only for networks with less than 5 genes, but also for larger networks with sparse

connectivities between genes. For example, Node 1 (Cln3) in Figure 1(B) is a pure "parent" node, which does not have any regulation coming from all other "children" nodes, i.e., all $w_{1j} = 0$ for $j \neq 1$, and for $\mathbf{S}_1$ the condition (6) does not hold:

$$\sum_{j \in J^+(\mathbf{S}_1)} w_{1j} - \sum_{j \in J^-(\mathbf{S}_1)} w_{1j} = -w_{11} \not\leq -\beta. \tag{9}$$

To avoid this problem, the factor β is introduced such that

$$W = \beta \hat{W}, \tag{10}$$

so to satisfy the condition (5,6), $\hat{w}_{ij}$ can only take values $[-1, 0, 1]$ without any restriction on the number of nonzero elements in each row of $\hat{W}$. For the sake of notational simplicity, in the sequel we still use W instead of $\hat{W}$, but write dynamics (2) as

$$\begin{aligned} S_i(t+\tau) &= \sigma(\sum_{j=1}^{n} w_{ij} S_j(t)) \\ &= \frac{2}{1 + \exp[-\beta \sum_{j=1}^{n} w_{ij} S_j(t)]} - 1. \end{aligned} \tag{11}$$

The necessary and sufficient condition for $\mathbf{S}$ to be a saturated equilibrium state for dynamics (11) then become

$$\sum_{j \in J^+(\mathbf{S})} w_{ij} - \sum_{j \in J^-(\mathbf{S})} w_{ij} \geq 1, \quad \text{if } i \in J^+(\mathbf{S}), \tag{12}$$

$$\sum_{j \in J^+(\mathbf{S})} w_{ij} - \sum_{j \in J^-(\mathbf{S})} w_{ij} \leq -1, \quad \text{if } i \in J^-(\mathbf{S}). \tag{13}$$

For saturated equilibrium $\mathbf{S}_1$, (12,13) may be written as

$$-\sum_{j=1, j\neq 5,9}^{11} w_{ij} \geq 1 - w_{i5} - w_{i9}, \quad \text{if } i = 5, 9, \tag{14}$$

$$-\sum_{j=1, j\neq 5,9}^{11} w_{ij} \leq -1 - w_{i5} - w_{i9}, \quad \text{if } i \neq 5, 9, \tag{15}$$

or

$$\sum_{j=1,j\neq 5,9}^{11} w_{ij} \leq -1 + w_{i5} + w_{i9}, \quad \text{if } i = 5,9, \tag{16}$$

$$\sum_{j=1,j\neq 5,9}^{11} w_{ij} \geq 1 + w_{i5} + w_{i9}, \quad \text{if } i \neq 5,9. \tag{17}$$

Condition (16,17) will be used to construct all viable networks W for dynamics (11) sharing saturated equilibrium state $\mathbf{S}_1$.

When w_{ij} takes values [-1,0,1],

$$w_{i5} + w_{i9} = -2, -1, 0, 1, 2,$$

for all 9 possible combinations of (w_{i5}, w_{i9})

$$(-1,-1), (-1,0), (0,-1), (-1,1), (1,-1), (0,0), (1,0), (0,1), (1,1),$$

we can determine all permitted row patterns satisfying (16,17) for the other nine w_{ij}'s in each combination of (w_{i5}, w_{i9}).

To avoid $-\mathbf{S}$ and $\mathbf{0}$ as an equilibrium state and fixed point, similarly, the modified dynamics

$$\begin{aligned} S_i(t+\tau) &= \sigma(\sum_{j=1}^{n} w_{ij} S_j(t)) \\ &= \frac{2}{1 + \exp[-\beta(\sum_{j=1}^{n} w_{ij} S_j(t) - \theta_i)]} - 1 \end{aligned} \tag{18}$$

is proposed. The necessary and sufficient condition to have $\mathbf{S}$ as a saturated equilibrium state for dynamics (18) is

$$\sum_{j\in J^+(\mathbf{S})} w_{ij} - \sum_{j\in J^-(\mathbf{S})} w_{ij} - \theta_i \geq 1, \quad \text{if } i \in J^+(\mathbf{S}), \tag{19}$$

$$\sum_{j\in J^+(\mathbf{S})} w_{ij} - \sum_{j\in J^-(\mathbf{S})} w_{ij} - \theta_i \leq -1, \quad \text{if } i \in J^-(\mathbf{S}), \tag{20}$$

and for $\mathbf{S}_1$ (19,20) becomes

$$
\begin{aligned}
-\sum_{j=1,j\neq 5,9}^{11} w_{ij} - \theta_i &\geq \quad 1 - w_{i5} - w_{i9}, \quad \text{if } i = 5,9, && (21)\\
-\sum_{j=1,j\neq 5,9}^{11} w_{ij} - \theta_i &\leq -1 - w_{i5} - w_{i9}, \quad \text{if } i \neq 5,9, && (22)
\end{aligned}
$$

or

$$
\begin{aligned}
\sum_{j=1,j\neq 5,9}^{11} w_{ij} + \theta_i &\leq -1 + w_{i5} + w_{i9}, \quad \text{if } i = 5,9, && (23)\\
\sum_{j=1,j\neq 5,9}^{11} w_{ij} + \theta_i &\geq \quad 1 + w_{i5} + w_{i9}, \quad \text{if } i \neq 5,9. && (24)
\end{aligned}
$$

Condition (23,24) can be used to construct all viable networks W for dynamics (18) with a given set of θ_i's sharing saturated equilibrium state $\mathbf{S}_1$. Since there is no unambiguous biological interpretation of the values of θ_i as $[-1,0,1]$ for w_{ij}, we will not construct all such viable networks here.

1.1 Determination of w_{ij} for $i = 5,9$ in dynamics (11)

We determine all permitted row patterns of w_{ij} for $i \in J^+(\mathbf{S}_1)$, i.e., $i = 5,9$.

1.1.1 Determination of $w_{ij}(j \neq 5,9)$ with $w_{i5} + w_{i9} = -2$

According to (16) we have

$$\sum_{j=1,j\neq 5,9}^{11} w_{ij} \leq -3. \qquad (25)$$

The summation on the lefthand side is a negative number ≤ -3. To satisfy this condition, three to nine $w_{ij}(j \neq 5,9)$ can be -1. All permitted row patterns are counted in Table 2.

Table 2. Permitted row patterns of $w_{ij}(j \neq 5, 9)$ for $w_{i5} + w_{i9} = -2$

Number of -1	Number of 0	Number of 1	Number of patterns	
			Formula	Number
3	6	0	C_9^3	84
4	5	0	C_9^4	
	4	1	$C_9^4 C_5^1$	756
5	4	0	C_9^5	
	3	1	$C_9^5 C_4^1$	
	2	2	$C_9^5 C_4^2$	1,386
6	3	0	C_9^6	
	2	1	$C_9^6 C_3^1$	
	1	2	$C_9^6 C_3^2$	
	0	3	C_9^6	672
7	2	0	C_9^7	
	1	1	$C_9^7 C_2^1$	
	0	2	C_9^7	144
8	1	0	C_9^8	
	0	1	C_9^8	18
9	0	0	C_9^9	1
Sum				3,061

1.1.2 Determination of $w_{ij}(j \neq 5, 9)$ with $w_{i5} + w_{i9} = -1$

According to (16) we have

$$\sum_{j=1, j\neq 5,9}^{11} w_{ij} \leq -2. \tag{26}$$

To satisfy this condition, two to nine w_{ij} can be -1. All permitted row patterns are given in Table 3.

Table 3. Permitted row patterns of $w_{ij}(j \neq 5, 9)$ for $w_{i5} + w_{i9} = -1$

Number of -1	Number of 0	Number of 1	Number of patterns	
			Formula	Number
2	7	0	C_9^2	36
3	6	0	C_9^3	
	5	1	$C_9^3 C_6^1$	588
4	5	0	C_9^4	
	4	1	$C_9^4 C_5^1$	
	3	2	$C_9^4 C_5^2$	2,016
5	4	0	C_9^5	
	3	1	$C_9^5 C_4^1$	
	2	2	$C_9^5 C_4^2$	
	1	3	$C_9^5 C_4^3$	630
6	3	0	C_9^6	
	2	1	$C_9^6 C_3^1$	
	1	2	$C_9^6 C_3^2$	
	0	3	C_9^6	672
7	2	0	C_9^7	
	1	1	$C_9^7 C_2^1$	
	0	2	C_9^7	144
8	1	0	C_9^8	
	0	1	C_9^8	18
9	0	0	C_9^9	1
sum				4,105

1.1.3 Determination of $w_{ij}(j \neq 5, 9)$ with $w_{i5} + w_{i9} = 0$

According to (16) we have

$$\sum_{j=1, j\neq 5,9}^{11} w_{ij} \leq -1. \tag{27}$$

To satisfy this condition, there may be one to nine w_{ij} being -1. All permitted row patterns are given in Table 4.

Table 4. Permitted row patterns of $w_{ij}(j \neq 5, 9)$ for $w_{i5} + w_{i9} = 0$

Number of -1	Number of 0	Number of 1	Number of patterns	
			Formula	Number
1	7	0	C_9^1	9
2	7	0	C_9^2	
	6	1	$C_9^2C_7^1$	288
3	6	0	C_9^3	
	5	1	$C_9^3C_6^1$	
	4	2	$C_9^3C_6^2$	1,848
4	5	0	C_9^4	
	4	1	$C_9^4C_5^1$	
	3	2	$C_9^4C_5^2$	
	2	3	$C_9^4C_5^3$	3,276
5	4	0	C_9^5	
	3	1	$C_9^5C_4^1$	
	2	2	$C_9^5C_4^2$	
	1	3	$C_9^5C_4^3$	
	0	4	C_9^5	2,016
6	3	0	C_9^6	
	2	1	$C_9^6C_3^1$	
	1	2	$C_9^6C_3^2$	
	0	3	C_9^6	672
7	2	0	C_9^7	
	1	1	$C_9^7C_2^1$	
	0	2	C_9^7	144
8	1	0	C_9^8	
	0	1	C_9^8	18
9	0	0	C_9^9	1
sum				8,272

1.1.4 Determination of $w_{ij}(j \neq 5, 9)$ with $w_{i5} + w_{i9} = 1$

According to (16) we have

$$\sum_{j=1, j\neq 5,9}^{11} w_{ij} \leq 0. \quad (28)$$

To satisfy this condition, nine w_{ij} may be all 0, or one to nine w_{ij} may be -1. All permitted row patterns are given in Table 5.

Table 5. Permitted row patterns of $w_{ij}(j \neq 5, 9)$ for $w_{i5} + w_{i9} = 1$

Number of -1	Number of 0	Number of 1	Number of patterns	
			Formula	Number
0	9	0	C_9^9	1
1	8	0	C_9^1	
	7	1	$C_9^1 C_8^1$	81
2	7	0	C_9^2	
	6	1	$C_9^2 C_7^1$	
	5	2	$C_9^2 C_7^2$	1,044
3	6	0	C_9^3	
	5	1	$C_9^3 C_6^1$	
	4	2	$C_9^3 C_6^2$	
	3	3	$C_9^3 C_6^3$	3,528
4	5	0	C_9^4	
	4	1	$C_9^4 C_5^1$	
	3	2	$C_9^4 C_5^2$	
	2	3	$C_9^4 C_5^3$	
	1	4	C_9^4	3,402
5	4	0	C_9^5	
	3	1	$C_9^5 C_4^1$	
	2	2	$C_9^5 C_4^2$	
	1	3	$C_9^5 C_4^3$	
	0	4	C_9^5	2,016
6	3	0	C_9^6	
	2	1	$C_9^6 C_3^1$	
	1	2	$C_9^6 C_3^2$	
	0	3	C_9^6	672
7	2	0	C_9^7	
	1	1	$C_9^7 C_2^1$	
	0	2	C_9^7	144
8	1	0	C_9^8	
	0	1	C_9^8	18
9	0	0	C_9^9	1
sum				10,907

1.1.5 Determination of $w_{ij}(j \neq 5, 9)$ with $w_{i5} + w_{i9} = 2$

According to (16) we have

$$\sum_{j=1, j\neq 5,9}^{11} w_{ij} \leq 1. \tag{29}$$

To satisfy this condition, one of nine w_{ij} may be 1, or all nine w_{ij} may be 0, or one to nine w_{ij} may be -1. All permitted row patterns are given in Table 6.

Table 6. Permitted row patterns of $w_{ij}(j \neq 5, 9)$ for $w_{i5} + w_{i9} = 2$

Number of -1	Number of 0	Number of 1	Number of patterns	
			Formula	Number
0	8	1	C_9^1	9
0	9	0	C_9^9	1
1	8	0	C_9^1	
	7	1	$C_9^1 C_8^1$	
	6	2	$C_9^1 C_8^2$	333
2	7	0	C_9^2	
	6	1	$C_9^2 C_7^1$	
	5	2	$C_9^2 C_7^2$	
	4	3	$C_9^2 C_7^3$	2,304
3	6	0	C_9^3	
	5	1	$C_9^3 C_6^1$	
	4	2	$C_9^3 C_6^2$	
	3	3	$C_9^3 C_6^3$	
	2	4	$C_9^3 C_6^4$	4,788
4	5	0	C_9^4	
	4	1	$C_9^4 C_5^1$	
	3	2	$C_9^4 C_5^2$	
	2	3	$C_9^4 C_5^3$	
	1	4	$C_9^4 C_5^4$	
	0	5	C_9^4	4,032
5	4	0	C_9^5	
	3	1	$C_9^5 C_4^1$	
	2	2	$C_9^5 C_4^2$	
	1	3	$C_9^5 C_4^3$	
	0	4	C_9^5	2,016
6	3	0	C_9^6	
	2	1	$C_9^6 C_3^1$	
	1	2	$C_9^6 C_3^2$	
	0	3	C_9^6	672
7	2	0	C_9^7	
	1	1	$C_9^7 C_2^1$	
	0	2	C_9^7	144
8	1	0	C_9^8	
	0	1	C_9^8	18
9	0	0	C_9^9	1
sum				14,318

Considering all possible nine choices for (w_{i5}, w_{i9}), the total number of permitted row patterns of w_{ij} for $i = 5, 9$ is

$$3,061 + 2 \times 4,105 + 3 \times 8,272 + 2 \times 10,907 + 14,318 = \mathbf{72,219}.$$

1.2 Determination of w_{ij} for $i \neq 5, 9$ in dynamics (11)

We then determine all permitted row patterns of w_{ij} for $i \in J^{-}(\mathbf{S}_1)$, i.e., $i \neq 5, 9$. For $i \neq 5, 9$, inequality (15)

$$- \sum_{j=1, j\neq 5,9} w_{ij} \;\; \leq \;\; -1 - w_{i5} - w_{i9}, \quad \text{if } i \neq 5, 9$$

needs to be satisfied. Condition (15) can be rewritten as

$$\sum_{j=1, j\neq 5,9} (-w_{ij}) \;\; \leq \;\; -1 + (-w_{i5}) + (-w_{i9}), \quad \text{if } i \neq 5, 9$$

which is the same as the inequality (16) used to determine w_{ij} for $i = 5, 9$, except that w_{ij} is replaces by $-w_{ij}$. Therefore, all the permitted row patterns of w_{ij} for $i = 5, 9$ multiplied by -1 are the permitted row patterns for $i \neq 5, 9$. Hence, the total number of permitted row patterns for each $i (i = 1 - 11)$ is the same, $72, 219$. Then, the total number of viable networks for dynamics (11) with $n = 11$ and sharing the saturated equilibrium state $\mathbf{S}_1$ (and $\mathbf{S}_2$) is

$$72,219^{11} \approx 2.7872 \times 10^{53}.$$

As shown below, according to Figure 1(B) of the yeast cell-cycle network, the first row of W is restricted to be

$$(\; 1 \;\; 0 \;\; 0 \;\; 0 \;\; 0 \;\; 0 \;\; 0 \;\; 0 \;\; 0 \;\; 0 \;\; 0 \;),$$

then the total number of viable networks of the budding yeast for dynamics (11) is

$$72,219^{10} \approx 3.8594 \times 10^{48}.$$

There are many choices of networks consistent with the experimental observations.

1.3 Construction of the yeast cell-cycle network W with dynamics (11)

According to the definition for the green and red arrows as well as the yellow loop given by Li et al., the network W_0 directly constructed from Figure 1(B) is

$$W_0 = \begin{bmatrix} -1 & 0 & 0 & 0 & 0 & 0 & 0 & 0 & 0 & 0 & 0 \\ 1 & 0 & 0 & 0 & 0 & 0 & 0 & 0 & 0 & -1 & 0 \\ 1 & 0 & 0 & 0 & 0 & 0 & 0 & 0 & 0 & -1 & 0 \\ 0 & 0 & 1 & -1 & 0 & 0 & 0 & 0 & 0 & 0 & 0 \\ 0 & 0 & 0 & -1 & 0 & 0 & 1 & -1 & 0 & -1 & 0 \\ 0 & 0 & 0 & 0 & 0 & -1 & 1 & 0 & 0 & -1 & 1 \\ 0 & 0 & 0 & 0 & 0 & 0 & -1 & 0 & 0 & 1 & 1 \\ 0 & 1 & 0 & 0 & 0 & 0 & -1 & 0 & -1 & 0 & 0 \\ 0 & 0 & 0 & -1 & 0 & 1 & 1 & -1 & 0 & -1 & 0 \\ 0 & 0 & 0 & 0 & -1 & 0 & -1 & 1 & -1 & 0 & 1 \\ 0 & 0 & 0 & 0 & 0 & 0 & 0 & 1 & 0 & 1 & -1 \end{bmatrix}. \tag{30}$$

W_0 does not satisfy condition (16,17) for any saturated state and does not have a saturated equilibrium state for dynamics (11). However, W_0 will be used as a starting point (considered as an experimental observation of the connections of the network) to construct networks with the saturated equilibrium expression state $\mathbf{S}_1$. The construction of networks reduces to satisfying condition (16, 17) or (23, 24) consistent as much as possible with the connectivities in the experimental observation.

1. Row 1 ($i = 1 \in J^-(\mathbf{S}_1)$)

Suppose that

$$(w_{15}, w_{19}) = (0, 0)$$

in W_0 is true, then condition (17) requires

$$\sum_{j=1, j\neq 5,9}^{11} w_{1j} \geq 1 + 0 + 0 = 1.$$

There are three choices:

1. Suppose that the information ($w_{1j}(j \neq 1)$ are all zero) given in Figure 1(B) is correct. Then the only choice is

$$w_{11} = 1.$$

2. If a biologist has observation that there exist activating regulations from other nodes to Clin3, then some appropriate $w_{1j}(j \neq 5, 9)$ can be set to 1.

3. If the information ($w_{1j}(j \neq 1)$ are all zero, and $w_{11} = -1$) given in Figure 1(B) is correct, then we can introduce a threshold parameter θ_1 in dynamics (18)

$$S_1(t+\tau) = \frac{2}{1 + \exp[-\beta(\sum_{j=1}^{n} w_{1j} S_j(t) - \theta_1)]} - 1, \tag{31}$$

   such that the condition (24)

$$w_{11} + \theta_1 = -1 + \theta_1 \geq 1$$

is satisfied.

We will introduce threshold parameters θ_i later; and we choose row 1 of W as

$$(\ 1\ \ 0\ \ 0\ \ 0\ \ 0\ \ 0\ \ 0\ \ 0\ \ 0\ \ 0\ \ 0\ ).$$

2. Row 2 ($i = 2 \in J^-(\mathbf{S}_1)$)

For $i = 2 \in J^-(\mathbf{S}_1)$, we also have $(w_{25}, w_{29}) = (0, 0)$ in W_0 and

$$\sum_{j=1, j\neq 5,9}^{11} w_{2j} \geq 1$$

should be satisfied. The summation on the lefthand side is zero for row 2 of W_0. So we need to add at least one 1 in any position $j \neq 5, 9$. Without any additional information about activation for node 2, we add 1 in $j = 2$, i.e., a "self activation regulation". The other way is to set w_{21} to be 2, but this is beyond our restriction $w_{ij} \in [-1, 0, 1]$. If a biologist has sufficient

information to prove such a choice, it may be adopted. Therefore, we choose row 2 as

$$(\begin{array}{ccccccccccc} 1 & 1 & 0 & 0 & 0 & 0 & 0 & 0 & 0 & -1 & 0 \end{array}).$$

3. Row 3 ($i = 3 \in J^-(\mathbf{S}_1)$)

The situation of row 3 is the same as row 2. We choose

$$(\begin{array}{ccccccccccc} 1 & 0 & 1 & 0 & 0 & 0 & 0 & 0 & 0 & -1 & 0 \end{array}).$$

4. Row 4 ($i = 4 \in J^-(\mathbf{S}_1)$)

Similar to rows 2 and 3, we need to add 1 in some $j \neq 5, 9$ in row 4 of W_0. As we do not have any additional information, we still add 1 in $j = 4$ which cancels -1 in W_0 to give row 4 as

$$(\begin{array}{ccccccccccc} 0 & 0 & 1 & 0 & 0 & 0 & 0 & 0 & 0 & 0 & 0 \end{array}).$$

5. Row 5 ($i = 5 \in J^+(\mathbf{S}_1)$)

With $(w_{55}, w_{59}) = (0, 0)$ row 5 in W_0

$$(\begin{array}{ccccccccccc} 0 & 0 & 0 & -1 & 0 & 0 & 1 & -1 & 0 & -1 & 0 \end{array}).$$

satisfies the condition

$$\sum_{j=1, j\neq 5,9}^{11} w_{5j} \leq -1,$$

so we keep it.

6. Row 6 ($i = 6 \in J^-(\mathbf{S}_1)$)

With $(w_{65}, w_{69}) = (0, 0)$ the condition

$$\sum_{j=1, j\neq 5,9}^{11} w_{6j} \geq 1$$

should be satisfied, but it is 0 for row 6 in W_0. We need to add at least 1 in $j \neq 5, 9$. Without any additional information, we add 1 in $j = 6$ which cancels -1 there to give

$$(\begin{array}{ccccccccccc} 0 & 0 & 0 & 0 & 0 & 0 & 1 & 0 & 0 & -1 & 1 \end{array}).$$

7. Row 7 ($i = 7 \in J^-(\mathbf{S}_1)$)

With $(w_{75}, w_{79}) = (0, 0)$ row 7 in W_0

$$(\begin{array}{ccccccccccc} 0 & 0 & 0 & 0 & 0 & 0 & -1 & 0 & 0 & 1 & 1 \end{array})$$

satisfies the condition

$$\sum_{j=1, j \neq 5,9}^{11} w_{7j} \geq 1,$$

and we keep it.

8. Row 8 ($i = 8 \in J^-(\mathbf{S}_1)$)

Since

$$w_{85} + w_{89} = 0 - 1 = -1,$$

row 8 should satisfy the condition

$$\sum_{j=1, j \neq 5,9}^{11} w_{8j} \geq 1 + w_{85} + w_{89} = 0,$$

but it is -1 for row 8 in W_0. We need to add at least one 1 in $j \neq 5, 9$. Comparing Figure 1(B) with 1(A), we see that node 8 is a combination of node Clb5,6 and node DNA Replication. There is a green arrow between the two nodes which was ignored after combination. To represent this green arrow we can add 1 at $j = 8$ as "auto activation regulation" to give one choice as

$$(\begin{array}{ccccccccccc} 0 & 1 & 0 & 0 & 0 & 0 & -1 & 1 & -1 & 0 & 0 \end{array}).$$

Moreover, there are bi-activating regulations between nodes Mcm1/SFF and Clb1,2, but only one green arrow from node Clb5,6 to node Clb1,2. There

is a possibility to have a green arrow back from node Clb1,2 to node Clb5,6. So we may have another choice for row 8 as

$$(\ 0 \quad 1 \quad 0 \quad 0 \quad 0 \quad 0 \quad -1 \quad 1 \quad -1 \quad 1 \quad 0 \ ).$$

9. Row 9 ($i = 9 \in J^+(\mathbf{S}_1)$)

With $(w_{95}, w_{99}) = (0, 0)$ row 9 in W_0

$$(\ 0 \quad 0 \quad 0 \quad -1 \quad 0 \quad 1 \quad 1 \quad -1 \quad 0 \quad -1 \quad 0 \ )$$

satisfies the condition

$$\sum_{j=1, j\neq 5,9}^{11} w_{9j} \leq -1,$$

so we keep it.

10. Row 10 ($i = 10 \in J^-(\mathbf{S}_1)$)

Since

$$w_{10,5} + w_{10,9} = -1 - 1 = -2,$$

row 10 should satisfy the condition

$$\sum_{j=1, j\neq 5,9}^{11} w_{10,j} \geq 1 + w_{10,5} + w_{10,9} = -1.$$

Row 10 in W_0

$$(\ 0 \quad 0 \quad 0 \quad 0 \quad -1 \quad 0 \quad -1 \quad 1 \quad -1 \quad 0 \quad 1 \ )$$

satisfies the condition, and we keep it.

11. Row 11 ($i = 11 \in J^-(\mathbf{S}_1)$)

With $(w_{11,5}, w_{11,9}) = (0, 0)$ row 11 in W_0

$$(\ 0 \quad 0 \quad 0 \quad 0 \quad 0 \quad 0 \quad 0 \quad 1 \quad 0 \quad 1 \quad -1 \ )$$

satisfies the condition

$$\sum_{j=1, j\neq 5,9}^{11} w_{11,j} \geq 1,$$

and we keep it.

This procedure can be extended to sharing more than one saturated equilibrium state.

Combining all choices of the 11 rows, we finally obtain two yeast cell-cycle networks for dynamics (11)

$$W_1 = \begin{bmatrix} 1 & 0 & 0 & 0 & 0 & 0 & 0 & 0 & 0 & 0 & 0 \\ 1 & 1 & 0 & 0 & 0 & 0 & 0 & 0 & 0 & -1 & 0 \\ 1 & 0 & 1 & 0 & 0 & 0 & 0 & 0 & 0 & -1 & 0 \\ 0 & 0 & 1 & 0 & 0 & 0 & 0 & 0 & 0 & 0 & 0 \\ 0 & 0 & 0 & -1 & 0 & 0 & 1 & -1 & 0 & -1 & 0 \\ 0 & 0 & 0 & 0 & 0 & 0 & 1 & 0 & 0 & -1 & 1 \\ 0 & 0 & 0 & 0 & 0 & 0 & -1 & 0 & 0 & 1 & 1 \\ 0 & 1 & 0 & 0 & 0 & 0 & -1 & 1 & -1 & 0 & 0 \\ 0 & 0 & 0 & -1 & 0 & 1 & 1 & -1 & 0 & -1 & 0 \\ 0 & 0 & 0 & 0 & -1 & 0 & -1 & 1 & -1 & 0 & 1 \\ 0 & 0 & 0 & 0 & 0 & 0 & 0 & 1 & 0 & 1 & -1 \end{bmatrix}, \qquad (32)$$

and

$$W_2 = \begin{bmatrix} 1 & 0 & 0 & 0 & 0 & 0 & 0 & 0 & 0 & 0 & 0 \\ 1 & 1 & 0 & 0 & 0 & 0 & 0 & 0 & 0 & -1 & 0 \\ 1 & 0 & 1 & 0 & 0 & 0 & 0 & 0 & 0 & -1 & 0 \\ 0 & 0 & 1 & 0 & 0 & 0 & 0 & 0 & 0 & 0 & 0 \\ 0 & 0 & 0 & -1 & 0 & 0 & 1 & -1 & 0 & -1 & 0 \\ 0 & 0 & 0 & 0 & 0 & 0 & 1 & 0 & 0 & -1 & 1 \\ 0 & 0 & 0 & 0 & 0 & 0 & -1 & 0 & 0 & 1 & 1 \\ 0 & 1 & 0 & 0 & 0 & 0 & -1 & 1 & -1 & 1 & 0 \\ 0 & 0 & 0 & -1 & 0 & 1 & 1 & -1 & 0 & -1 & 0 \\ 0 & 0 & 0 & 0 & -1 & 0 & -1 & 1 & -1 & 0 & 1 \\ 0 & 0 & 0 & 0 & 0 & 0 & 0 & 1 & 0 & 1 & -1 \end{bmatrix}. \qquad (33)$$

W_1 and W_2 differ only for $w_{8,10}$.

1.4 Determination of threshold θ_i for dynamics (18) with W_0

As discussed above, we can also use W_0 without any change, but introduce the threshold parameters

$$\Theta^T = (\ \theta_1 \ \ \theta_2 \ \ \theta_3 \ \ \theta_4 \ \ \theta_5 \ \ \theta_6 \ \ \theta_7 \ \ \theta_8 \ \ \theta_9 \ \ \theta_{10} \ \ \theta_{11} \ ) \qquad (34)$$

satisfying

$$\begin{aligned} \sum_{j=1, j\neq 5,9}^{11} w_{ij} + \theta_i &\geq -1 + w_{i5} + w_{i9}, \quad \text{if } i = 5, 9, & (35) \\ \sum_{j=1, j\neq 5,9}^{11} w_{ij} + \theta_i &\leq 1 + w_{i5} + w_{i9}, \quad \text{if } i \neq 5, 9. & (36) \end{aligned}$$

One choice with the smallest magnitudes for θ_i's

$$\Theta^T = (\ 2\ \ 1\ \ 1\ \ 1\ \ 0\ \ 1\ \ 0\ \ 0\ \ 0\ \ 0\ \ 0\ ) \quad (37)$$

is obtained by using

$$\begin{aligned} \sum_{j=1, j\neq 5,9}^{11} w_{ij} + \theta_i &= -1 + w_{i5} + w_{i9}, \quad \text{if } i = 5, 9, & (38) \\ \sum_{j=1, j\neq 5,9}^{11} w_{ij} + \theta_i &= 1 + w_{i5} + w_{i9}, \quad \text{if } i \neq 5, 9. & (39) \end{aligned}$$

2 Saturated equilibrium expression states for constructed networks

The saturated equilibrium expression states for a given network W in dynamics (11) can be determined by using the modified condition in Theorem 1

$$S_i \left(\sum_{j=1}^{n} w_{ij} S_j \right) \geq 1, \quad (i = 1, 2, \ldots, n). \quad (40)$$

For $n = 11$, there are $2^{11} = 2,048$ saturated states. All of the 2,048 states were tested by condition (40) for W_1 and W_2, respectively, to determine which of them are saturated equilibrium states of W_1 and W_2. The test for 2,048 states took only **0.01** seconds by Matlab on a Dell Precision Workstation T3400.

The saturated equilibrium expression states for a given network W with threshold vector Θ in dynamics (18) can be determined by using the condition

$$S_i \left(\sum_{j=1}^{n} w_{ij} S_j - \theta_i \right) \geq 1, \quad (i = 1, 2, \ldots, n). \quad (41)$$

The resultant saturated equilibrium expression states for W_1, W_2 and W_0 with Θ have been obtained as shown below.

1. W_1

W_1 has two saturated equilibrium expression states for dynamics (11)

$$\begin{aligned}\mathbf{S}_1 &= (\; -1 \; -1 \; -1 \; -1 \; 1 \; -1 \; -1 \; -1 \; 1 \; -1 \; -1 \;), \\ \mathbf{S}_2 &= (\; 1 \; 1 \; 1 \; 1 \; -1 \; 1 \; 1 \; 1 \; -1 \; 1 \; 1 \;)\end{aligned}$$

with

$$\mathbf{S}_2 = -\mathbf{S}_1.$$

2. W_2

W_2 has four saturated equilibrium expression states for dynamics (11)

$$\begin{aligned}\mathbf{S}_1 &= (\; -1 \; -1 \; -1 \; -1 \; 1 \; -1 \; -1 \; -1 \; 1 \; -1 \; -1 \;), \\ \mathbf{S}_2 &= (\; 1 \; 1 \; 1 \; 1 \; -1 \; 1 \; 1 \; 1 \; -1 \; 1 \; 1 \;), \\ \mathbf{S}_3 &= (\; -1 \; 1 \; -1 \; -1 \; 1 \; -1 \; -1 \; -1 \; 1 \; -1 \; -1 \;), \\ \mathbf{S}_4 &= (\; 1 \; -1 \; 1 \; 1 \; -1 \; 1 \; 1 \; 1 \; -1 \; 1 \; 1 \;).\end{aligned}$$

with

$$\mathbf{S}_2 = -\mathbf{S}_1, \qquad \mathbf{S}_4 = -\mathbf{S}_3.$$

The $\mathbf{S}_1$ and $\mathbf{S}_3$ are just the 1st and 3rd fixed point attractors in Table 1.

3. W_0 **with** Θ

For W_0 with parameters Θ, there is only a single saturated equilibrium state for dynamics (18)

$$\mathbf{S}_1 = (\; -1 \; -1 \; -1 \; -1 \; 1 \; -1 \; -1 \; -1 \; 1 \; -1 \; -1 \;). \tag{42}$$

3 Robustness to noise

First, the numbers of saturated initial expression states converging to each equilibrium expression state for W_1, W_2 are determined by either directly solving the dynamics (11) or using the modified condition of Theorem 5

$$\begin{aligned}
\beta(\sum_{j=1}^{n} w_{ij} S_j(t \geq k)) &> -\ln[(\alpha_i - 1) - \sqrt{(\alpha_i - 1)^2 - 1}], i \in J^+(\mathbf{S}), \\
\beta(\sum_{j=1}^{n} w_{ij} S_j(t \geq k)) &< \ln[(\alpha_i - 1) - \sqrt{(\alpha_i - 1)^2 - 1}], i \in J^-(\mathbf{S}).
\end{aligned}$$

For W_1, the CPU times are **0.8** and **0.3** seconds, respectively to check all 2,048 saturated states, i.e., using Theorem 5 the CPU time is $\sim 41\%$ of that for direct solving the sigmoidal function. The results are given in Table 7.

Table 7. The number of saturated initial expression states converging to different equilibrium states for different gene networks

Final state	Number of saturated initial states		
	W_0 with Θ	W_1	W_2
$\mathbf{S}_1$	2,048	1,024	979
$\mathbf{S}_2$		1,024	979
$\mathbf{S}_3$			45
$\mathbf{S}_4$			45

The robustness to noise R_{n_t} is defined as

$$R_{n_t} = 1/m$$

where m is the total number of fixed points. The robustness to noise R_{n_i} of a given saturated equilibrium expression state $\mathbf{S}_i$ for a gene network W is specified by the ratio of the number N_i of saturated initial expression states converging to $\mathbf{S}_i$, with respect to the total number 2^n of possible saturated initial states

$$R_{n_i} = N_i/2^n.$$

Note that for W_1, W_2, no saturated initial state converges to the unstable fixed point $\mathbf{0}$. Therefore, in the calculation of R_{n_t}, we ignore $\mathbf{0}$ and only consider the saturated equilibrium states. The robustness to noise R_{n_t} and

R_{n_i} are given in Table 8. There are significant differences between $R_{n_i}(i = 1, 2, 3, 4)$ for W_2. Obviously, the saturated equilibrium states $\mathbf{S}_1, \mathbf{S}_2$ are much more stable than $\mathbf{S}_3, \mathbf{S}_4$.

Table 8. The Robustness to noise R_{n_t} and R_{n_i} for different gene networks

Network	R_{n_t}	R_{n_i}			
		$\mathbf{S}_1$	$\mathbf{S}_2$	$\mathbf{S}_3$	$\mathbf{S}_4$
W_0 with Θ	1	1			
W_1	1/2	1/2	1/2		
W_2	1/4	0.478	0.478	0.022	0.022

The robustness to noise $R_{n_{ij}}$ of a given viable pair of saturated equilibrium and initial expression states $\mathbf{S}_i$ and $\mathbf{S}_j(0)$ for a gene network W is specified by the ratio of the number N_{ij} of neighboring saturated initial expression states differing from $\mathbf{S}_j(0)$ by only one element and still converging to $\mathbf{S}_i$, with respect to the total number n of possible one element differing saturated initial states

$$R_{n_{ij}} = N_{ij}/n.$$

The robustness to noise $R_{n_{ij}}$ for each viable pair of saturated equilibrium and initial expression states was calculated. The distribution of $R_{n_{ij}}$, i.e., how many pairs with the same value of $R_{n_{ij}}$, is given in Tables 9 and 10.

Table 9. The distribution of $R_{n_{ij}}$ for the networks W_0 with Θ and W_1

Final state	$R_{n_{ij}}$ (for W_0 with Θ)	$R_{n_{ij}}$ (for W_1)
	11/11	10/11
$\mathbf{S}_1$	2,048	1,024
$\mathbf{S}_2$		1,024

Table 10. The distribution of $R_{n_{ij}}$ for the network W_2

Final state	$R_{n_{ij}}$								
	2/11	3/11	4/11	5/11	6/11	7/11	8/11	9/11	10/11
$\mathbf{S}_1$					1	12	29	146	791
$\mathbf{S}_2$					1	12	29	146	791
$\mathbf{S}_3$	1	6	21	7	3	7			
$\mathbf{S}_4$	1	6	21	7	3	7			

The results show that W_0 with Θ is completely stable for any viable pair; for W_1, there is one neighbour of $\mathbf{S}_j(0)$ differing at the first element, which

causes a change in the saturated equilibrium state $\mathbf{S}_i$; for W_2, the distribution of $R_{n_{ij}}$ is divergent, and $\mathbf{S}_1$ and $\mathbf{S}_2$ are much more stable than $\mathbf{S}_3$ and $\mathbf{S}_4$.

4 Robustness to mutation

The robustness to mutation R_{m_i} for saturated equilibrium state $\mathbf{S}_i$ is defined as

$$R_{m_i} = \frac{N_W^v}{N_W} = \sum_{i=1}^{n} \frac{N_{w_i}^v}{2n^2},$$

where N_W^v and $N_{w_i}^v$ respectively are the total numbers of viable (keeping the saturated equilibrium state unchanged) single w_{ij} changes of W and its ith row; $N_W(= 242)$ is the total number of possible single w_{ij} changes of W. R_{m_i}'s have been calculated for W_1, W_2 and W_0 with the Θ given above as shown in Table 11. Note that for $\mathbf{S}_1$ the robustness to mutation R_{m_i} is almost the same for W_1, W_2 and W_0 with Θ.

Table 11. The robustness to mutation R_{m_i} of W_0 with Θ, W_1 and W_2

Final state	W_0 with Θ		W_1		W_2	
	N_W^v	R_{m_i}	N_W^v	R_{m_i}	N_W^v	R_{m_i}
$\mathbf{S}_1$	137	0.57	140	0.58	143	0.59
$\mathbf{S}_2$			140	0.58	143	0.59
$\mathbf{S}_3$					131	0.54
$\mathbf{S}_4$					131	0.54

Robustness to mutation $R_{m_{ij}}$ for a specified pair of saturated equilibrium and initial states $\mathbf{S}_i$ and $\mathbf{S}_j(0)$ of a viable network W is defined as

$$R_{m_{ij}} = \frac{N_{m_{ij}}^v}{N_W} = \frac{N_{m_{ij}}^v}{2n^2},$$

where $N_{m_{ij}}^v$ is the total number of viable single w_{ij} changes of W with respect to a specified states $\mathbf{S}_i$ and $\mathbf{S}_j(0)$. $N_{m_{ij}}^v$ can be obtained by determining how many networks in N_W^v, which share the saturated equilibrium state $\mathbf{S}_i$, also share the saturated initial expression state $\mathbf{S}_j(0)$. The resultant distribution, i.e., how many viable pairs having the same $R_{m_{ij}}$ for W_1, W_2 and W_0 with the Θ given above is shown in Table 12 and Figure 2.

Table 12. The distribution of $R_{m_{ij}}$ for W_1, W_2 and W_0 with Θ

$R_{m_{ij}}$	W_0 with Θ	W_1	W_2	
	$\mathbf{S}_1$	$\mathbf{S}_1$ and $\mathbf{S}_2$	$\mathbf{S}_1$ and $\mathbf{S}_2$	$\mathbf{S}_3$ and $\mathbf{S}_4$
94/242			2	
95/242			3	
96/242			5	
97/242			14	
98/242			5	
99/242			7	
100/242			10	
101/242			5	
102/242			2	
103/242	2			
104/242	4		1	1
105/242	7			
106/242	7		1	
107/242	7			
108/242	9		1	
109/242	7	5	1	1
110/242	8	14	1	1
111/242	10	13		
112/242	8	10		
113/242	10	5		1
114/242	9			1
115/242	6	1		2
116/242	2	2		4
117/242	4	3		2
118/242	3	1		5
119/242	6	4	1	4
120/242	6	2	8	1
121/242	2	6	4	3
122/242	6	13	5	6
123/242	5	18	1	1
124/242	8	9	6	7
125/242	39	11	8	1
126/242	93	18	10	2
127/242	86	22	29	
128/242	123	38	49	1
129/242	133	71	56	
130/242	180	117	50	
131/242	212	114	36	1
132/242	234	113	77	
133/242	206	132	95	
134/242	183	129	85	
135/242	212	75	69	
136/242	145	52	85	
137/242	66	13	75	
138/242		4	75	
139/242		8	43	
140/242		1	25	
141/242			16	
142/242			10	
143/242			3	
sum	2,048	1,024	979	45

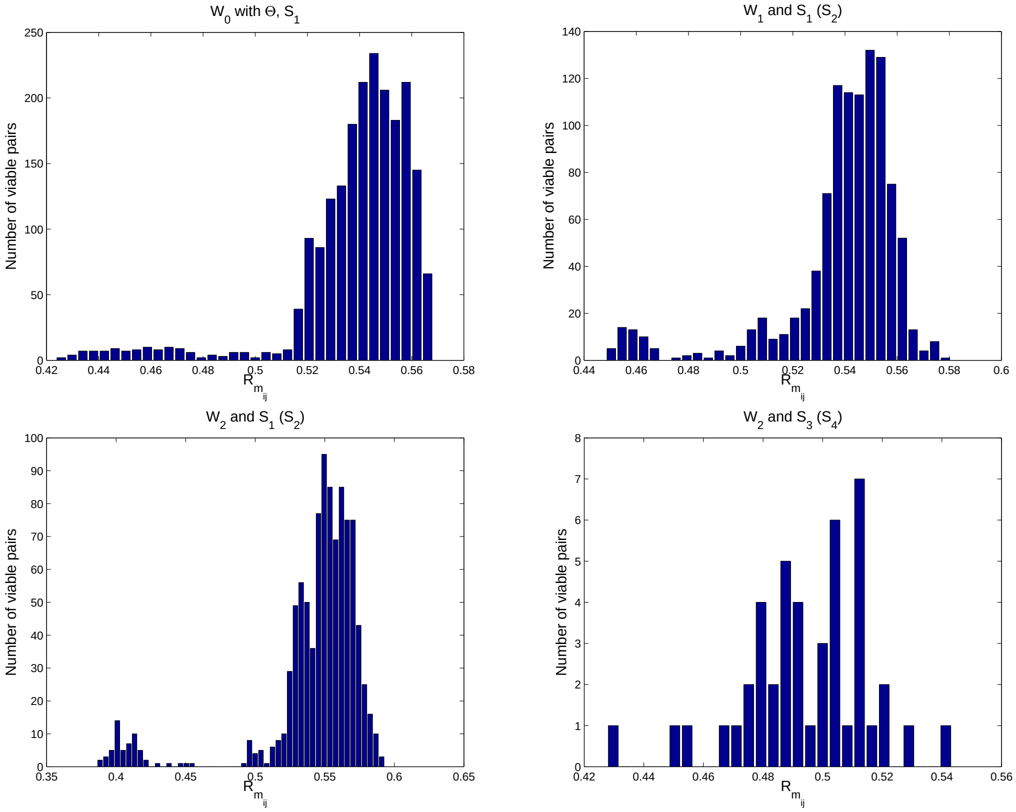

Figure 2: Distribution of $R_{m_{ij}}$ for W_1, W_2 and W_0 with the Θ.
